# Polymeric and Polymer-Functionalized Drug Delivery Vectors: From Molecular Architecture and Elasticity to Cellular Uptake

**DOI:** 10.3390/polym17162243

**Published:** 2025-08-19

**Authors:** Thorsten Auth

**Affiliations:** Theoretical Physics of Living Matter, Institute for Advanced Simulation, Forschungszentrum Jülich, 52425 Jülich, Germany; t.auth@fz-juelich.de

**Keywords:** linear chains, star polymers, polymer-grafted nanoparticles, hairy nanoparticles, stealth liposomes, nanogels, microgels, biomolecular condensates, circulation times, cellular uptake, translocation, passive endocytosis, drug delivery

## Abstract

Polymers and polymer composites offer versatile possibilities for engineering the physico-chemical properties of materials on micro- and macroscopic scales. This review provides an overview of polymeric and polymer-decorated particles that can serve as drug-delivery vectors: linear polymers, star polymers, diblock-copolymer micelles, polymer-grafted nanoparticles, polymersomes, stealth liposomes, microgels, and biomolecular condensates. The physico-chemical interactions between the delivery vectors and biological cells range from chemical interactions on the molecular scale to deformation energies on the particle scale. The focus of this review is on the structure and elastic properties of these particles, as well as their circulation in blood and cellular uptake. Furthermore, the effects of polymer decoration in vivo (e.g., of glycosylated plasma membranes, cortical cytoskeletal networks, and naturally occurring condensates) on drug delivery are discussed.

## 1. Introduction

Polymeric materials are very versatile with regard to their elastic properties and surface functionalization. They can be readily produced at the laboratory scale, but the processes can be scaled up to the industrial level. Furthermore, polymers are routinely used to functionalize surfaces and equip them with the desired properties. Because of their versatility, polymer materials and composites are also important candidates for engineering vectors for targeted drug delivery. Synthetic polymeric molecules and particles for drug delivery can come in very different flavors: as linear chains, star polymers, diblock-copolymer micelles, polymer-grafted nanoparticles, polymer-decorated vesicles, polymersomes, nano- and microgels, and biomolecular condensates. Two further polymeric architectures used for drug delivery, capsules and dendrimers, are not discussed in the following but have been reviewed recently in specialized articles [1,2,3].

**Figure 1:**
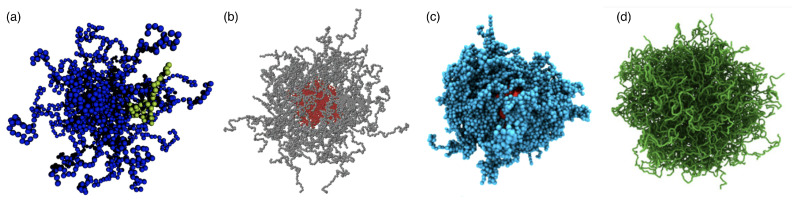
Simulation snapshots of polymeric particles. (**a**) Star polymer with f=35 arms and $N_{s}=50$ monomers (dark, blue beads) per chain and a linear chain with $N_{c}=40$ monomers (bright, green beads) at a center-to-center distance of ten times the bead radius. Reprinted with permission from Ref. [8]. Copyright 2007 American Chemical Society. (**b**) Diblock-copolymer micelle. Reprinted with permission from Ref. [9]. Copyright 2006 American Chemical Society. (**c**) Polymer-grafted nanoparticle. Reprinted with permission from Ref. [10]. Copyright 2020 American Chemical Society. (**d**) Swollen microgel particle with a uniform crosslink distribution. Reprinted with permission from Ref. [11].

Each architecture of polymeric drug-delivery vectors comes with specific structural and elastic properties. For example, linear chains in a good solvent have, on average, an elongated shape and experience strong shape fluctuations. In contrast, stars with high functionalities, micelles, hairy particles with a high polymer density, and microgels can be thought of as spherical soft colloids (see Figure 1). Typical sizes of linear chains and star polymers for drug delivery are in the range of tens of nanometers, whereas biomolecular condensates, stealth liposomes, polymersomes, and microgels can reach sizes of a micrometer and more (see Table 1). Consequently, the interactions between particles with different architectures and biological cells can be fundamentally different. Whereas linear chains with suitable hydrophilic and hydrophobic properties translocate through lipid-bilayer membranes, soft-spherical colloids are likely to be wrapped by the membranes. Elasticity, a key parameter to characterize polymeric materials, has also been identified as a key parameter for the cellular uptake of particles [4,5,6,7].

For larger polymeric particles, an atomistic resolution is not the appropriate approach to capture the relevant physics and chemistry. Beyond the scale of single monomers, linear polymers are often described as (semi-)flexible chains with persistence length $\ell_{p}$ (see Table 2). The persistence length characterizes the decay of the orientational correlation function $\langle e(s)\cdot e(s+\Delta s)\rangle =\exp\left[-\Delta s/\ell_{p}\right]$ along the chain, where e is a tangential unit vector and *s* a coordinate along the contour. From a calculation point of view, also the model of freely hinged chains is convenient, where *N* straight rod-like segments with Kuhn length $\ell_{K}$ are linked to each other at vertices to form a polymer with total contour length $\ell_{c}=N\ell_{K}$. Both, persistence and Kuhn length, characterize the stiffness of linear polymer chains and are closely related, $\ell_{K}=2\ell_{p}$ [33]. Whole-chain properties, such as the chain’s root mean square end-to-end distance

(1) $$R_{e}=\sqrt{\langle R_{e2e}^{2}\rangle }=\ell_{K}N^{1/2}$$

with $R_{e2e}=\left|r_{N}-r_{0}\right|$ the difference between the position vectors of the last and the first vertices, and the root mean square radius of gyration

(2) $$R_{g}=\sqrt{\langle R_{\mathrm{gyr}}^{2}\rangle }=\frac{R_{e}}{\sqrt{6}}$$

with $R_{\mathrm{gyr}}^{2}=\left(1/N\right)\sum_{i=0}^{N}{\left(r_{i}-\langle r_{i}\rangle \right)}^{2}=\left(1/\left(2N^{2}\right)\right)\sum_{i=0}^{N}\sum_{j=0}^{N}{\left(r_{i}-r_{j}\right)}^{2}$, here for freely-hinged chains, fluctuate subject to thermal fluctuations of the polymer conformations. Synthetic polymers are often very flexible with persistence lengths in the nanometer range. Linear polymers with N≫1 act as entropic springs with a spring constant

(3) $$k_{\mathrm{sp}}=\frac{3k_{B}T}{R_{e}^{2}}$$

and can exert an entropic pressure leading to an increase of the free energy, e.g.,

(4) $$F_{\mathrm{conf}}=k_{B}TN{\left(\frac{\ell_{K}}{d}\right)}^{5/3}$$

if polymers in good solvent are confined to a tube of diameter *d* [34]. The bulk modulus of many polymeric delivery vectors consisting of flexible chains at low densities is therefore of the order of a few $k_{B}T/{\left(100\;\mathrm{nm}\right)}^{3}\approx \left(10-100\right)\;\mathrm{Pa}$. Cytoskeletal filaments, on the contrary, have persistence lengths in the micrometer range and above, and thus form strong scaffolds for biological cells.

Lipid-bilayer membranes, often supported by a cortical cytoskeleton, are the barriers and communication interfaces that separate the interior of biological cells from their environment. Thus, understanding the interaction of elastic particles with lipid-bilayer membranes is crucial for designing drug-delivery vectors. Using polymeric materials and polymer composites, not only can the molecular architectures, sizes, shapes, and elasticities be conveniently engineered, but also the molecular properties such as hydrophobicity/hydrophilicity, electrical charge, and surface functionalization with ligands. For example, the hydrophobic–hydrophilic sequence of their monomers affects the interaction of polymers with the hydrophilic headgroups and the hydrophobic tails of the lipids in bilayers. Additionally, a surface functionalization with polymers that contain charged or zwitterionic groups can significantly alter the interaction of delivery vectors with lipid-bilayer membranes (e.g., positively charged monomers are attracted to typically negatively charged cell plasma membranes [48,49]). Whereas hydrophobic and electrostatic interactions can mediate attraction between polymers and lipids, the steric interaction between polymer-functionalized surfaces induces repulsion [50,51,52]. The molecular properties of polymeric and polymer-functionalized delivery vectors can also be used to control the attachment of a protein corona when they are exposed to biological fluids. Controlling the formation of a corona is key for engineering the interactions of the vectors with lipids, proteins, and ligands in the plasma membranes of cells [53,54].

Polymers sensitive to external stimuli, such as temperature, pH, and light, may undergo externally triggered transitions that can be exploited for both drug loading and release. For example, polymers that change their solubility depending on the pH value of the buffer may disrupt lysosomes upon acidification and deliver their cargo to the cytosol [55]. Polymers with protonatable groups may promote endosomal escape via a proton sponge effect [56].

Linear polymers deliver drugs across plasma membranes when their design balances key physicochemical attributes. For example, a moderate cationic charge density facilitates membrane binding and cellular uptake when distributed evenly along the polymer chain [57]. However, depending on the number and arrangement of cations, positively charged polymers can also induce cytotoxicity [58,59]. Hydrophobic modifications have been found to enhance interactions with lipid bilayers and allow polymers to translocate through membranes [60,61,62,63]. However, hydrophobic polymers can lead to pore formation in bilayers and may thus induce cytotoxicity [64]. Therefore, effective drug delivery by linear polymers relies on molecular fine-tuning of the (i) molecular weight, (ii) backbone persistence length, (iii) hydrophobic modifications, and (iv) charges.

Star polymers with optimized physico-chemical properties can cross plasma membranes effectively; multifunctional star polymers are delivered to cells in vitro in less than 15 min [65]. Star polymers have been successfully applied for the non-viral delivery siRNA that forms a complex with the arms of the star [66,67]. Furthermore, star polymers are taken up using dynamin-dependent clathrin-mediated endocytosis by cancerous cells [68]. Star polymers can also be used for retarded drug delivery: nanostars that accumulate in endosomes of spinal neurons have been shown to release a drug against pain over a period of 24 h [69].

Diblock-copolymer micelles can incorporate hydrophilic cargo, such as siRNA, in their outer shells [70], and hydrophobic drugs, such as doxorubicin, in their cores [71]; pH sensitivity can help to release the drugs [72]. At higher concentrations, multi-micellar aggregates may co-exist with single micelles. Single micelles and aggregates can interact differently with biological cells because of their size difference [73,74,75]. For diblock-copolymer micelles, tuning the molecular architecture has a two-fold role: optimizing and targeting cellular uptake and drug loading. An appropriately tuned hydrophilic–hydrophobic balance and suitable lengths of the hydrophobic and hydrophilic blocks are key from the point of view of drug loading.

Polymer-grafted (“hairy”) nanoparticles (PGNs) are particles that are decorated with densely grafted linear polymers. This includes particles with low-functionality stars self-assembled around cargo, similar to the self-assembly of diblock-copolymer micelles [71,72]. A polymer coat can add surface functionalization and drug storage to magnetic nanoparticles [76], and can equip mesoporous silica nanoparticles with ultrasound-responsive drug-release properties [77]. Furthermore, thanks to the particle core, polymer-grafted nanoparticles enable us to synthesise delivery vectors with a wide range of sizes. PGNs can be engineered with small sizes of tens of nanometers suitable for clathrin-mediated endocytosis [78], but also with sizes of several micrometers suitable for phagocytic uptake [79,80]. Particles functionalized with polymers can show prolonged circulation times in the blood compared with bare particles [81]. For PEG-coated gold nanoparticles, it has been shown that an increased molecular weight of the PEG can increase the circulation time by an order of magnitude [82].

Polymersomes often consist of diblock-copolymer bilayers, analogously to liposomes consisting of lipid bilayers [83]. However, their membranes are usually significantly thicker and thus more rigid and stable than their lipid-bilayer counterparts. Therefore, conventional polymersomes have a low permeability of their membranes [84]. More recently, advanced polymersomes with membrane thicknesses similar to those of liposomes show higher permeabilities [85]; this also applies to polyion complex vesicles that consist of an electrostatically complexed shell of charged polyelectrolytes [86]. In conventional polymersomes, hydrophilic drugs can be incorporated in the aqueous core [87], and hydrophobic drugs in the hydrophobic layer of the membrane [88]. Recently, thermoplasmonic polymersomes containing small gold nanoparticles have been shown to induce death of cancer cells in vitro through collective heating [89].

Stealth liposomes are liposomes coated with polymers that lead to prolonged circulation times and reduced macrophage uptake. The liposomes are traditionally modified using PEG. In addition to the steric repulsion, the polymer layer can also lead to measurable differences in surface charge [90] and hydrophilicity [91]. An increased molecular weight of the PEG molecules used for surface modification can increase the hydrodynamic thickness of the polymer coat of the liposomes and decrease their zeta potential [92]. Furthermore, a PEG coating of liposomes can lead to a decreased drug-release rate [93].

Nano- and microgels have emerged as versatile materials for biological applications, ranging from drug delivery to tissue engineering [94]. Their physicochemical properties are tunable with respect to a wide range of materials, sizes, shapes, and elastic moduli. Nano- and microgels have the ability to encapsulate both hydrophilic and hydrophobic drugs [95,96]. Microgels can be designed to interact with peptides and proteins, making them suitable for macromolecular drug delivery [97]. Injectable microgel–hydrogel composites have shown prolonged small-molecule drug release [93]. The 3D crosslinked polymer networks of the gels allow us to engineer microgels with homogeneous elasticity, but also with core-shell or hollow architectures [98]. Polymeric gels are also used as cores for lipid nanoparticles to modify the liposome elasticity [99,100,101]. Recently, ultralow-crosslinked (ULC) microgels have received increased attention due to their special elastic and flow properties. When solubilized in a droplet that dries at room temperature, swollen ULC microgels do not show a coffee-ring effect [102], and in flow they behave as hard or soft objects depending on their concentration [103]. ULC microgels can pass through capillaries smaller than their diameter [104], can be incorporated within a fibrin network without restricting cell motility [105], and have been shown to collapse on a fibrin network mimicking behavior known from platelets in blood clotting in vivo [106].

Biomolecular condensates, also referred to as coacervates, can accommodate small molecules, proteins, nucleic acids, enzymes, and substrates. Synthetic condensates, inspired by their in vivo counterparts, have thus been recognized as drug reservoirs and developed to serve as drug-delivery vectors. They can deliver both hydrophilic drugs, such as antibodies, which individually do not easily cross the hydrophobic core of the cell membrane [107], and hydrophobic drugs that are not easily solubilized in water [108]. Condensation occurs below an upper or above a lower critical solution temperature (UCST, LCST) [109,110]; condensate sizes range from tens of nanometers to hundreds of micrometers and can be tuned by, for example, temperature [111] and pH [70]. The stimulus sensitivity of biomolecular condensates can be exploited, e.g., for endosomal escape. Cellular uptake processes for biomolecular condensates include active cellular uptake by macropinocytosis and phagocytosis [112], as well as direct cytosolic delivery [113,114,115], such that condensates used for drug delivery as large as ≈1μm can be taken up by cells in the presence of endocytosis inhibitors [112,114]. The properties of biomolecular condensates can be engineered by polymer concentration and chain length, but also by the chemical properties of the polymers and the cargo. Condensates can be coated with lipids to modify their interactions with lipid-bilayer membranes [107,116].

In the following, the structural and elastic properties, circulation times in the blood, and cellular uptake of polymeric and polymer-functionalized drug-delivery vectors will be discussed in more detail. Three main cellular uptake mechanisms, translocation, passive endocytosis, and active uptake, will be reviewed. Finally, a summary and an outlook will be provided.

## 2. Structural and Elastic Characterization

The architecture of polymeric and polymer-functionalized materials depends both on the type of polymeric material and its molecular architecture. Here, the long-standing history of polymer research offers a range of modeling approaches, spanning from Flory theory and blob models to more recent analytical methods. The models for structure and deformation energy presented below allow us to characterize the deformability of the polymeric particles, which is key to calculating their elastic deformations upon interaction with the plasma membranes of cells and for constructing models for cellular uptake.

### 2.1. Linear Polymers

**Figure 2:**
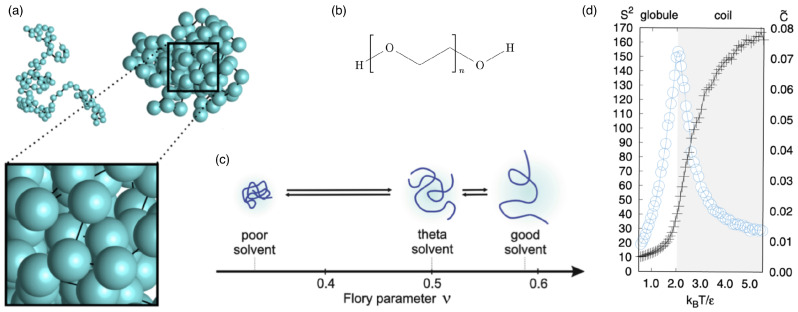
Linear polymers. (**a**) Typical configurations of a linear chain with N=102 monomers in the coil state and a liquid-like globular state. Reprinted with permission from Ref. [119]. Copyright 2017 by the American Physical Society. (**b**) Chemical structure of polyethylene glycol (PEG). (**c**) Shapes and Flory parameters ν of polymer chains in different solvent qualities. The three dotted lines denote the theoretical values of ν for different solvent qualities. Reproduced from Ref. [121] with permission from Springer Nature. (**d**) Reduced mean square radius of gyration $S^{2}={\left(R_{g}/\ell_{K}\right)}^{2}$ (plus symbols in the left scale) and mean-squared deviation of the total energy from its average $\tilde{C}$ (proportional to the specific heat; circles in the right scale) as functions of the reduced temperature $k_{B}T/\epsilon$, determined from the Monte Carlo simulations of Gaussian chains for N=102. Reprinted with permission from Ref. [119]. Copyright 2017 by the American Physical Society.

Linear polymers are the basic building blocks of many polymeric materials (see Figure 2). On the atomic scale, the detailed chemical structure best defines the interactions within and between molecules; on the scale of the entire molecule, persistence length (discussed earlier) and solvent quality are often the appropriate concepts to characterize the properties of linear chains. If chains with contour lengths much larger than the persistence length are considered, three states can be distinguished depending on the solvent quality: (i) in bad solvents, the polymer collapses and forms a compact globule; (ii) in so-called “theta solvent,” the polymer behaves like an ideal random walk; and (iii) in good solvents, the polymer swells beyond the ideal random walk and typically has an elongated shape [117]. In bad solvents, the monomers are densely packed, and the interactions are dominated by the attractive and the excluded-volume interactions between the monomers. Consequently, the size of the polymer globule scales as $N^{1/3}$ with the number of monomers. So-called theta solvents are defined such that the properties of linear chains are described by Equations (1)–(3). In good solvents, the root mean square end-to-end distance of linear chains has been shown to scale as

(5) $$R_{e}\propto N^{\nu }\;,$$

where ν is the Flory exponent, which has been estimated analytically to be ν=3/5 and numerically to be ν=0.588 [118].

Whereas many properties of ideal random walks for linear polymers in theta solvents can be accurately calculated analytically, self-avoiding linear chains are often studied using computer simulations (see Figure 2a). One simulation model is the freely hinged chain with Gaussian bond-length distribution and Lennard–Jones beads at the vertices that ensure self-avoidance. If the ratio of the bead size σ to the equilibrium bond length $\ell_{K}$ is carefully chosen, it allows the formation of a liquid state at low temperatures [119,120]. For potential depth ϵ, the attractive interaction potential between the beads leads to a collapsed liquid-like globular conformation of the polymer chain for temperatures $T≲2\epsilon /k_{B}$ and a to random coil for $T≳2\epsilon /k_{B}$. At the transition, the radius of gyration $R_{g}$ increases sharply, and the specific heat, characterized by the mean-squared deviation of the total energy from its average $\tilde{C}=\left[\langle E_{\mathrm{int}}^{2}\rangle -{\langle E_{\mathrm{int}}\rangle }^{2}\right]/\left(N^{2}\epsilon^{2}\right)$, shows a peak (see Figure 2d).

### 2.2. Star Polymers

**Figure 3:**
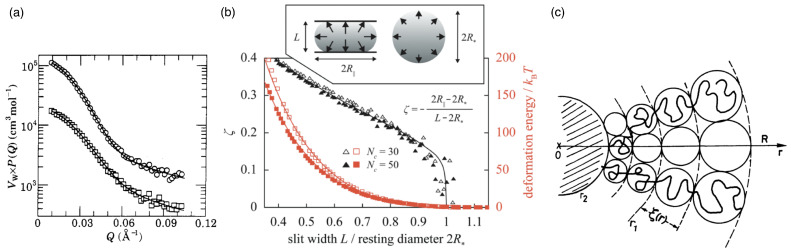
Star polymers. (**a**) Form factors and corresponding fits (solid lines) of the 8-arm (squares) and 18-arm (circles) polyisoprene stars (8-arm data are multiplied by a factor of 0.3 for visibility). Figure reproduced from Ref. [124]. Published under licence by IOP Publishing Ltd. (**b**) Liquid-drop model. Reduced central lateral extension ζ of a diametrically compressed spherical polymer brush with f=60 arms containing $N_{c}=30$ and 50 monomers plotted against slit width *L* (black datapoints/curve). The datapoints are obtained using molecular dynamics simulations, and the curve is the liquid drop model fit for the reduced Egelstaff–Widom length Ψ=0.6 (see Equation (8)). The deformation energies for these two cases, together with the fits (red datapoints/curves), are also shown. The inset illustrates the diametral-compression geometry, with arrows representing hydrostatic pressure, and contains the definition of ζ. Figure reproduced from Ref. [122]. CC BY-NC 3.0. (**c**) A representation of the Daoud–Cotton model: every branch is made of a succession of blobs with a size ξ increasing from the centre of the star to the outside. From outside to inside, $r_{1}$ indicates the transition between the swollen and the unswollen region, and $r_{2}$ the transition between the unswollen region and the core. The terms swollen, unswollen, and core refer to regions with increasing monomer concentration. Figure reprinted from Ref. [125].

Star polymers consist of *f* linear chains attached to each other at their ends to form a star, where *f* is the functionality of the star. Stars with low functionality are dominated by the nature of the linear chains, which is also seen by the smeared-out scattering intensity (see Figure 3a), while stars with high functionality resemble spherical soft colloids. In the liquid drop model for star polymers, the arms are considered fluid because they can readily interchange their positions. The free energy of the star polymer reads [122]

(6) $$F=\chi_{T}^{-1}\left(V-V_{0}-V_{0}\ln\frac{V}{V_{0}}\right)+\gamma_{F}A_{F}+\frac{1}{2}\gamma_{C}A_{C}\;,$$

with $\chi_{T}$ being the isothermal compressibility at the reference volume $V_{0}$ at which the pressure in the drop vanishes, *V* the actual drop volume, $\gamma_{F}$ the tension of the drop–solvent interface, and $A_{F}$ the drop–solvent interface area. For star-polymer aggregates, the terms with interfacial tension $\gamma_{C}\neq \gamma_{F}$ at the contact zones, the contact area $A_{C}$ between stars, and the accordingly reduced drop–solvent interface area $A_{F}$ are taken into account for aggregate formation. For calculating the total contact area in star-polymer aggregates, the interfacial area is the area shared by two drops and should be considered only once.

Because the tension of the drop–solvent interface compresses a free star polymer, its equilibrium volume $V_{*}<V_{0}$ is smaller than the reference volume; the resting volume $V_{*}<V_{0}$ is found by minimizing the energy of an isolated drop. To the lowest order, the relative volume decrease of free stars is given by the product $\Pi_{L}\chi_{T}$, where

(7) $$\Pi_{L}=\frac{2\gamma_{F}}{R_{0}}$$

is the osmotic pressure for the resting drop radius $R_{*}<R_{0}={\left(3V_{0}/4\pi \right)}^{1/3}$. The compressibility $\chi_{T}$ is determined via the Egelstaff–Widom length

(8) $$\Psi =\frac{2\gamma_{F}\chi_{T}}{R_{0}}\;,$$

below which molecular physics is required to model liquids and above which macroscopic laws can be employed [123]. At high *f*, the liquid drops are incompressible and their equilibrium volumes are similar to the resting volume, $V_{*}\approx V_{0}$, which corresponds to Ψ→0. If the equilibrium volume $V_{*}$ is much smaller than the resting volume $V_{0}$, then Ψ≫1. For deformations of the star smaller than the Egelstaff–Widom length, the liquid drop model allows predicting the lateral extension and the deformation energy upon compresssion between two parallel walls (see Figure 3b); the deformation energy change is orders of magnitude larger than the thermal energy $k_{B}T$.

Star polymers can alternatively be modeled using a blob model [125] (see Figure 3c). Each arm of the star is subdivided into blobs with energy $\approx k_{B}T$ each, whose sizes are determined by the sterical hindrance by monomers from the other arms. Therefore, the blob diameter $\xi \left(r\right)\propto rf^{-1/2}$ increases linearly with the distance *r* from the center of the star. The confinement of the arm motion requires smaller blobs near the center and larger blobs towards the surface of the star. The total energy is the sum of the elastic energy

(9) $$F_{\mathrm{el}}\propto \frac{R_{s}^{2}}{N_{f}f^{1/2}\ell_{K}^{2}}k_{B}T\;,$$

and the interaction energy

(10) $$F_{\mathrm{int}}\propto {\left(N_{f}f^{1/2}\right)}^{2}\frac{vf^{-1/2}}{R_{s}^{3}}k_{B}T$$

over all arms of the star, $F_{\mathrm{star}}=\sum_{i=1}^{f}\left(F_{\mathrm{el}}+F_{\mathrm{int}}\right)$. Here, $R_{s}$ is the radius of the star polymer, $N_{f}$ the number of segments per arm, and *v* is the excluded volume associated with a segment.

### 2.3. Polymer-Grafted Nanoparticles

**Figure 4:**
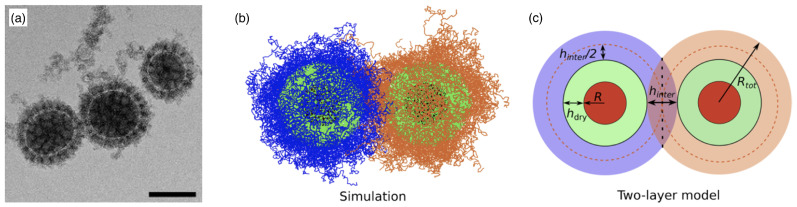
Polymer-grafted nanoparticles. (**a**) Cryo-electron micrograph of mixed poly(acrylic acid)/polystyrene brush-grafted silica nanoparticles in water. The scale bar corresponds to 100nm. Reprinted with permission from Ref. [22]. Copyright 2015 American Chemical Society. (**b**,**c**) Simulation snapshot and schematic representation of the two-layer model. Indicated are the nanoparticle radius *R*, the total radius of the polymer-grafted nanoparticle $R_{\mathrm{tot}}$, and the thicknesses of the dry and interpenetration layers, $h_{\mathrm{dry}}$ and $h_{\mathrm{inter}}$, respectively. Reprinted with permission from Ref. [10]. Copyright 2020 American Chemical Society.

Polymer-grafted nanoparticles (PGNs) are spherical hard particles decorated with a dense layer of linear, end-grafted polymer chains (see Figure 4a). In melts, the two-layer model assumes that polymeric chains of neighbouring polymer-grafted nanoparticles interpenetrate in an outer layer of thickness $h_{\mathrm{inter}}$ and are expelled by the chains of the grafted nanoparticle in an inner layer of thickness $h_{\mathrm{dry}}$ (see Figure 4b,c). For calculating the interaction energy between two PGNs, the analytical model developed to calculate $h_{\mathrm{inter}}$ and $h_{\mathrm{dry}}$ in polymer melts agrees well with the computer simulations. The model may also be applied to estimate the deformation energy for single PGNs in solution [10], although in this case a more rigorous approach is the blob model for a brush on a hard core similar to the model discussed for star polymers. In the two-layer model, the height of the polymer brush is

(11) $$h={\left(R_{\mathrm{core}}^{3}+\frac{3ZN_{c}}{4\pi \rho }\right)}^{1/3}-R_{\mathrm{core}}\;$$

for a core radius $R_{\mathrm{core}}$, grafting density $\rho_{g}$ and number $Z=4\pi R_{c}^{2}\rho_{g}$, and the segment volume number density ρ. This leads to the chain extension free energy

(12) $$E_{\mathrm{ext}}=\frac{3k_{B}Th^{2}}{2N_{c}\ell_{K}^{2}}=\frac{3k_{B}T}{2N_{c}\ell_{K}^{2}}{\left[{\left(R_{\mathrm{core}}^{3}+\frac{3ZN_{c}}{4\pi \rho }\right)}^{1/3}-R_{\mathrm{core}}\right]}^{2}\;.$$

The energy simplifies in the limit of a large core to the energy

(13) $$E_{\mathrm{ext}}^{\mathrm{pln}}\approx \frac{3k_{B}T}{2}{\left(\frac{Z}{4\pi \rho R_{\mathrm{core}}^{2}\ell_{K}}\right)}^{2}N$$

for a brush anchored to a planar substrate, and in the ‘star-polymer’ limit of a small core to

(14) $$E_{\mathrm{ext}}^{\mathrm{sph}}\approx \frac{3k_{B}T}{2}{\left(\frac{3Z}{4\pi \rho \ell_{K}^{3}}\right)}^{2/3}N^{-1/3}\;.$$

### 2.4. Polymersomes and Stealth Liposomes

**Figure 5:**
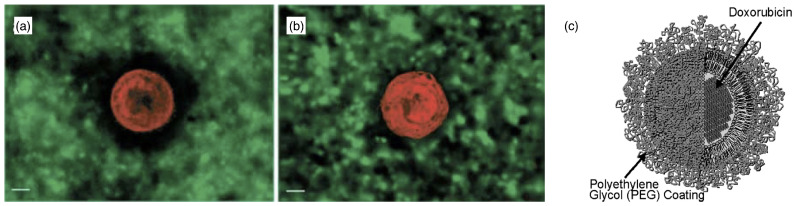
Polymer-decorated membranes. 3D reconstruction of the pericellular hyaluronan coat by particle exclusion assay. (**a**,**b**) Fluorescence micrographs of rhodamine-labeled chondrocytes immersed in a medium containing fluorescein isothiocyanate (FITC)-labeled silica beads. Cells were allowed to adhere to glass coverslips for 25min before fixation and labeled with tetramethyl rhodamine isothiocyanate (red). They were then incubated with FITC-labeled 0.4μm silica beads (green). Micrographs were taken with a digital microscope (DeltaVision) able to generate 3D images by image reconstruction from a series of z-sections at 0.5μm resolution. The excluded volume is dark. Untreated cells have a 5 to 6μm wide excluded zone around them (**a**), whereas beads reach up to the surface of hyaluronidase-treated cells (**b**). The scale bars correspond to 5μm. Reprinted from Ref. [126], with permission from the Biophysical Society. (**c**) Pegylated liposomal doxorubicin. Reprinted from Ref. [127], Copyright 2004, with permission from Elsevier.

Polymersomes and stealth liposomes are built using polymer-decorated membranes. Polymer functionalization of membranes is a common motif both in vivo and for synthetic drug-delivery vectors, used to regulate the interaction with their environments (see Figure 5). Prominent examples are chondrocytes that produce a thick coat of hyaluronan [126], and polymer-coated stealth liposomes with improved circulation times in blood flow [127]. At sparse coverage, the linear polymers end-grafted to membranes assume mushroom shapes, which act as spacers that induce steric repulsion [128] and locally deform the membranes [129,130,131,132]. Because of the entropic repulsion between the polymer chains and the membranes, despite being in the mushroom regime, polymer-decorated membranes are stiffer than bare membranes [133,134,135,136]. However, for drug-delivery applications, a dense polymer coat is more effective to prevent a vector from interacting with other surfaces than sparsely distributed polymer mushrooms. Beyond stealth liposomes, many conventional polymersomes can be rationalized as formed by a polymer-brush-coated elastic surface [137], although some recent polymersomes, and also dendrimersomes, have thin membranes similar to lipid bilayers [85,138].

The steric repulsion due to the polymer coat of stealth liposomes and polymersomes may be modeled similarly to PGNs. The effective curvature-elastic properties of polymer-decorated membranes can be rationalized using a continuum-membrane model [139],

(15) $$E_{\mathrm{def}}=\int \;dS\left[2\kappa {\left(H-c_{0}\right)}^{2}+\bar{\kappa }K\right]\;,$$

with the integral calculated over the total membrane area *S*. Here, the membrane shape in each point is described by the mean curvature *H* and the Gaussian curvature *K*. The curvature-elastic parameters describe the membrane’s material properties, with κ the bending rigidity, $c_{0}$ the spontaneous curvature, and $\bar{\kappa }$ the Gaussian saddle-splay modulus. The effective curvature-elastic constants of polymer-decorated membranes can be considered through a modification of their values for bare membranes $\kappa_{\mathrm{eff}}=\kappa +\Delta \kappa$, $c_{0,\mathrm{eff}}=c_{0}+\Delta c_{0}$, and ${\bar{\kappa }}_{\mathrm{eff}}=\bar{\kappa }+\Delta \bar{\kappa }$. For sparse polymer decoration, in the mushroom regime [133,134,135,136],

(16) $$\frac{\Delta \kappa }{k_{B}T}=a_{\kappa }\sigma_{p}R_{e}^{2}\;\frac{\kappa_{\mathrm{eff}}}{k_{B}T}\Delta c_{0}=a_{\mathrm{sp}}\sigma_{p}R_{e}\;\frac{\Delta \bar{\kappa }}{k_{B}T}={\bar{a}}_{\kappa }\sigma_{p}R_{e}^{2}$$

for surface density $\sigma_{p}$ of end-grafted linear, ideal polymer chains, $a_{\kappa }=0.21$, $a_{\mathrm{sp}}=0.18$, and ${\bar{a}}_{\kappa }=-0.17$. In the brush regime, the curvature-elastic constants of polymer-decorated membranes are dominated by the polymer brush [136],

(17) $$\begin{array}{ccc} \frac{\Delta \kappa }{k_{B}T} & = & \frac{\nu +2}{12\nu^{2}}R_{e}^{6}\ell_{K}^{3\nu -6}\sigma_{p}^{3\nu /2}\\ \frac{\kappa_{\mathrm{eff}}}{k_{B}T}\Delta c_{0} & = & \frac{1}{8\nu }R_{e}^{4}\ell_{K}^{2/\nu -4}\sigma_{p}^{\left(2+\nu )/(2\nu \right)}\\ \frac{\Delta \bar{\kappa }}{k_{B}T} & = & -\frac{1}{6\nu }R_{e}^{6}\ell_{K}^{3\nu -6}\sigma_{p}^{3\nu /2}\;, \end{array}$$

with ν=3/5.

### 2.5. Nano- and Microgels

**Figure 6:**
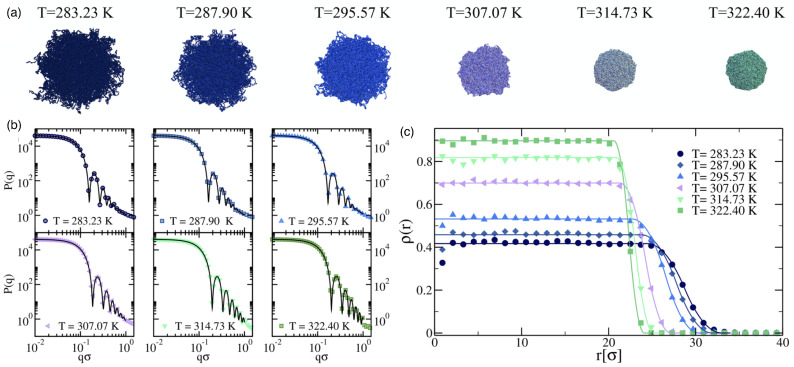
Microgel collapse: in silico modeling and scattering experiments. (**a**) Snapshots of a microgel particle, consisting of crosslinked linear chains with beads that interact using the Weeks–Chandler–Andersen (WCA) potential for ’bead size’ σ, exhibiting the typical volume phase transition from swollen to compact. (**b**) Numerical form factors, averaged over four different realizations, for microgels with N≈ 41,000 monomers and c=3.2% of crosslinkers generated in a sphere of radius Z=30σ (symbols) for various solvent qualities (corresponding to temperatures). Solid lines are fits of the curves using the fuzzy sphere model of Equation (18). (**c**) Averaged density profiles obtained from molecular dynamics (MD) simulations of a microgel with N≈ 41,000, c=3.2%, and Z=30σ (symbols) and from the fit of the form factors using the fuzzy sphere model (solid lines). Reprinted with permission from Ref. [140].

**Figure 7:**
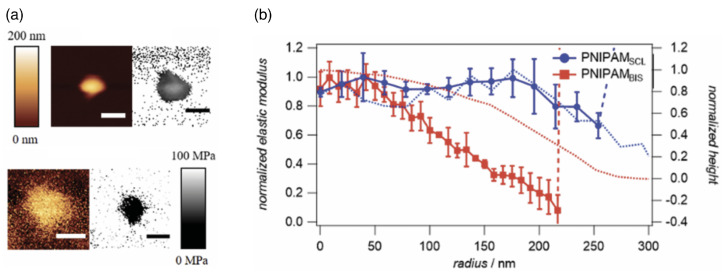
The elastic moduli of deposited ${\mathrm{PNIPAM}}_{\mathrm{BIS}}$ microgels that are crosslinked using N,N’-methylenebis(acrylamide) (BIS) and of ${\mathrm{PNIPAM}}_{\mathrm{SCL}}$ self-crosslinked microgels as a function of the radial position. (**a**) Typical topography and elastic modulus images of a single PNIPAM microgel (top: BIS, bottom: SCL). Scale bars: 200 nm. (**b**) Plot of the elastic modulus normalized by the elastic modulus at the centre, $Y_{c}=340\pm 10\;\mathrm{kPa}$ for ${\mathrm{PNIPAM}}_{\mathrm{BIS}}$ and $Y_{c}=13\pm 3\;\mathrm{kPa}$ for ${\mathrm{PNIPAM}}_{\mathrm{SCL}}$, vs. the radial position calculated from at least five microgels using high-resolution elastic modulus mapping and the height trace (dashed lines) reconstructed from vertical tip position during force map acquisition. Reprinted from Ref. [142]. CC BY 3.0.

Nano- and microgels are polymeric particles with a 3D network whose architecture can be controlled during synthesis. The gels have fixed connections between their building blocks and thus a finite shear modulus. Many microgels are stimuli-responsive microparticles that swell and collapse depending on the temperature and/or pH [140,141], which considerably changes both their sizes and elastic properties (see Figure 6 and Figure 7). If the temperature of a PNIPAM microgel is increased above the volume phase transition temperature (VPTT) ≈34 °C of the microgel, which for PNIPAM is similar to the lower critical solution temperature (LCST) ≈32 °C of the linear polymers, the microgels collapse due to the removal of the hydration layer surrounding the polymer chains and the formation of polymer–polymer contacts. Typical swelling ratios for PNIPAM are between 2 and 4, depending on the type of the crosslinkers [142]. Using scattering experiments and computer simulations, the fuzzy sphere model with a radially symmetrical density [140,143,144,145]

(18) $$\frac{\rho (r)}{\rho_{0}}=\left\{\begin{array}{cc} 1 & \mathrm{if}\;r<R_{c}\\ 1-\frac{{\left(r-R^{\prime }+2\sigma_{\mathrm{surf}}\right)}^{2}}{8\sigma_{\mathrm{surf}}^{2}} & \mathrm{if}\;R_{c}\leq r<R^{\prime }\\ \frac{{\left(R^{\prime }-r+2\sigma_{\mathrm{surf}}\right)}^{2}}{8\sigma_{\mathrm{surf}}^{2}} & \mathrm{if}\;R^{\prime }\leq r<R^{\prime \prime }\\ 0 & \mathrm{if}\;r\geq R^{\prime \prime } \end{array}\right.$$

has been developed, which is capable of describing the normalized radial polymer density profile of stimuli-sensitive microgels at various temperatures. Here, $\rho_{0}$ is the polymer density in the inner core, and the smearing parameter $\sigma_{\mathrm{surf}}$ corresponds to about half the thickness of the fuzzy shell; $R_{c}=R^{\prime }-2\sigma_{\mathrm{surf}}$ is the radius of the inner sphere of the microgel with a constant monomer density, $R^{\prime }$ is usually referred to as the core radius, and $R^{\prime \prime }=R^{\prime }+2\sigma_{\mathrm{surf}}$ is the total radius including the fuzzy shell.

The elastic moduli of microgels can be routinely determined using atomic force microscopy (AFM) for surface-adhered microgels [32,146,147] (see Figure 7); various other techniques for characterizing the mechanical properties of polymeric particles have been recently reviewed in Ref. [148]. Radial stiffness measurements for microgels adsorbed to a planar substrate using AFM show an increasing Young’s modulus toward the center/apex of the gels [32,142]. The decrease of the measured elasticities towards the rim of the microgel is usually interpreted as higher polymer and crosslinker densities in the core and lower densities in the outer shell [140]. Therefore, microgels have been proposed to mutually interact in a brush-like manner with a polymeric corona surrounding a dense core [149]. Table 3 shows the wide range of elastic moduli that have been measured in the center of microgels with radii relevant to drug-delivery applications, which can be engineered during synthesis [150]; ultra-low crosslinked microgels have very small elastic moduli and do not show a radial stiffness gradient [142,151,152]. Swollen microgels in good solvents are often modeled as homogeneous elastic spherical particles with Young’s moduli *Y* proportional to the number of crosslinks, and can be simulated using networks of harmonic springs [153]. The Poisson’s ratio of swollen microgels is $\nu_{P}=0.25$, due to the central forces between the crosslinks [154]. An increase in temperature leads to a decrease in solvent quality and therefore to a collapse of the microgel [32]. In the collapsed state, computer simulations using bead-spring models for the polymers show that with increasing temperature, the Poisson’s ratio initially decreases with decreasing solvent quality and finally assumes values $0.4<\nu_{P}<0.5$ in the collapsed state [155]. A microgel in the collapsed state has a significantly higher Young’s modulus compared with the swollen state (see Table 3) and may thus in some systems also be modeled as a spherical hard particle [156,157].

**Figure 8:**
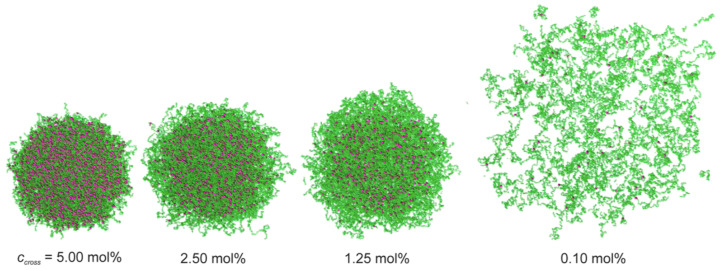
Representative snapshots of in silico microgels down to ultra-low crosslinked for microgels with N≈ 336,000 monomers interacting via the WCA potential for ‘bead size’ σ in good solvent conditions, mimicking T=20 °C in experiments. To improve the visualization, all the plots represent a slice of the microgels of width 30σ. Monomers are reported in green, while crosslinkers are in red. For ultra-low crosslinked (ULC) microgels, we use $c_{\mathrm{cross}}=0.1\%$, which agrees well with the experimental data in terms of swelling ratio and form factors. Reprinted with permission from Ref. [151]. Copyright 2023 American Chemical Society.

Ultra-low crosslinked (ULC) ultrasoft microgels were first reported in 1993 for PNIPAM [160] (see Figure 8). Subsequently, the effect of self-crosslinking has been studied—expanding on the previous understanding of the phase behaviour of PNIPAM [161,162]—to understand the origin of the stability of these self-crosslinked (SCL) microgels that form without additional crosslinkers [163,164,165,166]. The size of ULC microgels can be controlled, e.g., by the addition of a surfactant during precipitation polymerization [167]. In their swollen state, ULC microgels have homogeneous Young’s moduli ≈10kPa. Because of their softness, they easily attach and spread on substrates [104,152,168,169]. ULC PNIPAM microgels with radii of more than 200nm have been found to easily translocate through pores with radii of 50nm [104]. In peak-force tapping mode, sharp AFM tips experience very low forces of the order of 100pN, and the microgels become invisible to the AFM [170]. Upon collapse, deswelling can lead—depending on size, ionic, and electrostatic interactions—to internal microphase separation [171,172]. Compared with microgels of similar collapsed radii ≈150 nm and synthesized at finite crosslinker concentrations, which have hydrodynamic radii of 300–350 nm in their swollen states, ULC microgels swell to 400–550 nm [151].

The 3D network architecture makes microgels an especially versatile class of polymeric particles. Variations of conventionally crosslinked microgels with an approximately homogeneous distribution of polymers include core-shell microgels [173], core-double-shell microgels [174], core-shell microgels with anisotropic shapes [175], and hollow microgels [176]. Microgels with several shells may have several temperatures for swollen–collapsed transitions [174]. Hollow, pH-sensitive microgels can, for example, serve as stimuli-responsive nanocontainers for hydrophilic drugs [176]. Ultra-low crosslinked microgels can be synthesized by penetrating and enclosing a core formed by a conventionally crosslinked microgel [177,178,179].

### 2.6. Biomolecular Condensates

Biomolecular condensates are lyophilic colloids formed by the condensation of macromolecules via liquid–liquid phase separation (LLPS), which is often driven by electrostatic interactions [180]. An equilibrium mean-field theory approach for the gelation of associative polymers that interact via “stickers” (e.g., ionic or hydrophobic groups) via reversible junctions is always accompanied by a tendency of the system to phase-separate [181]. Because the condensates lack a lipid-bilayer membrane bounding them, such gel-like colloids are sometimes also referred to as membrane-less organelles or droplets [182]. Condensates are often small, with sizes between a few and a few hundred nanometers in living cells [18], and can reach micrometer sizes outside cells. In vivo, the condensates form and dissolve depending on their environment and dynamically structure the cytoplasm, as also shown in the seminal study on *C. elegans* [183]; the physical processes determining the sizes of the condensates are often still unclear. An exponential size distribution has been found for nuclear speckles whose growth is determined by initially fast and then gradual coalescence, whereas Huntingtin aggregates with a continuous material production are power-law distributed [184]. In vitro, the size distribution of the condensates can be controlled, e.g., by the polymer concentration in the systems in which the condensates are formed and the temperature [111], or by the composition in multi-component condensates [185].

**Figure 9:**
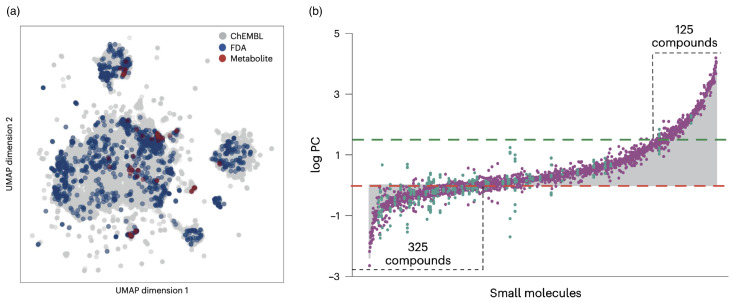
Biomolecular condensates for delivering small molecules. (**a**) Uniform manifold approximation and projection (UMAP) representation of 1700 small molecules used in the analysis [187], which is based on physical features generated in QikProp [188]. (**b**) Partition coefficients (PC) determined using mass spectrometry vary over nearly six orders of magnitude. Bar chart of PC values, ordered from smallest to largest, for the partitioning of 1037 compounds into the SUMOSIM condensate (composed of polySUMO and polySUMO-interaction motif (SIM) proteins [189]). Red and green dashed lines indicate logPC=0 and $\log{\mathrm{PC}}_{\mathrm{SUMOSIM}}=1.77$, respectively. The numbers of compounds with logPC<0 and $\log\mathrm{PC}>\log{\mathrm{PC}}_{\mathrm{SUMOSIM}}$ are indicated. The grey-coloured areas represent the bar plots for the mean values of the data, and green and purple dots represent metabolites and drug compounds, respectively. Reprinted from Ref. [186]. CC BY 4.0.

Biomolecular condensates are unique as potential drug-delivery vectors because they can accommodate a wide variety of molecules and help deliver them to cells. Partitioning small molecules into condensates with folded domains has recently been studied for ≈1700 biologically relevant small molecules, including ≈200 metabolite compounds and ≈1500 drug compounds approved by the US Food and Drug Administration (FDA), complemented by ≈100,000 randomly chosen biologically acrive small molecules from the ChEMBL database [186]. The pharmacokinetic, physicochemical, and chemical properties have been predicted computationally, and a dimension reduction technique showed that the selection of the biologically relevant molecules is distributed evenly across the chemical space of the random components (see Figure 9a). The partition coefficients span a range of $10^{6}$, from exclusion to enrichment in the condensates (see Figure 9b). The results suggest that physical properties, such as hydrophobicity, are more important for the partitioning than specific chemical structures. More diverse condensate systems remain to be tested.

**Figure 10:**
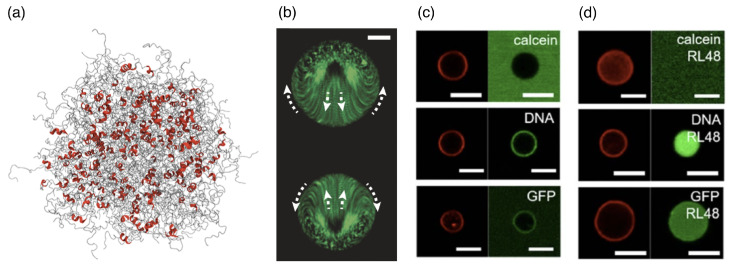
Biomolecular condensates containing proteins. (**a**) Intrinsically disordered proteins (IDPs) mediate phase separation that underlies the formation of a biomolecular condensate. Reprinted with permission from Ref. [194]. Copyright 2024 American Chemical Society. (**b**) Two chemically active protein condensates, pinned to a planar glass substrate functionalized with polyethylene glycol diacrylate with $M_{n}=700$ (PEGDA 700) at a distance compared to the protein diameter, show internal flows induced by the presence of the other condensate. Time projection over 1min of fluorescent particles inside two adjacent chemically active protein droplets catalyzing the urea-urease reaction (overall enzyme concentration $c_{e}=0.6\;\mu M$, substrate concentration $c_{s=100\;\mathrm{mM}}$). Arrows indicate the internal flow direction. The scale bar corresponds to 10μm. Reprinted from Ref. [199]. CC BY 4.0. (**c**,**d**) Permeability of giant unilamellar vesicles (GUV) and lipid-coated protein condensates (RL48 protocells). A dye compound, calcein (20μM), 21 base pair DNA (0.5μM), and green fluorescent protein (GFP) are treated to (**c**) GUV and (**d**) RL48 protocells for 10min before confocal analysis. Lipid coatings (red) and dye-labeled external materials (green) are imaged. The scale bars correspond to 5μm. Reprinted from Ref. [116]. CC BY-NC 3.0.

In a “stickers” picture, for proteins, DNA, and RNA, specific chemical interactions may serve as stickers, with the chemical nature of the spacer region in-between changing the behavior of a condensate from fluid to solid. Understanding the rules that govern the interactions between small molecules and the biomolecules within the condensates is also referred to as “chemical grammar” [190]. DNA itself can serve as a base material to hierarchically design condensates for delivering cargo to cells [191]. A partitioning of large cargo into condensates can also be achieved through specific interactions, as demonstrated for biotin-functionalized beads [192]; moreover, beads with a specific surface chemistry can be incorporated into immiscible condensates. Intrinsically disordered regions of proteins are often involved in binding to other molecules and inducing the formation of a condensate [193,194] (see Figure 10a). Using hybrid-resolution computer simulations that consider local structural motifs, the importance of β-sheet structures for interactions has been predicted [194]. However, an overall charge can also drive the partitioning of larger molecules into biomolecular condensates [192,195,196], with specific binding between their amino or nucleic acids and other components of the condensate only modulating a multiphase condensation [189,197]. Intriguingly, engineered biomolecular condensates containing enzymes or other chemically active components are non-equilibrium systems that can show internal flows and active regulation of their sizes [198,199]. Such non-equilibrium processes have been studied using large condensates (see Figure 10b) but may also be relevant for the smaller condensate sizes relevant to drug delivery. Interestingly, biomolecular condensates readily exchange molecules with their environment even in the presence of lipid coating—unlike vesicles whose intact lipid-bilayer membranes are impenetrable to many components (see Figure 10c,d).

The mechanical properties of colloids can range from fluid to solid-like [200]. For fluid condensates, typical interface tensions between the condensates and water/cytosol are usually small, in the range between 1 and 100μN/m, and in some cases they can also reach 1mN/m (see Table 4). A tension of the order of 1μN/m has also been predicted theoretically for colloid–polymer mixtures [201]. For comparison, oil–water and air–water interface tensions are of the order of 10–100 mN/m. Experimentally, the interface tensions can be determined using a variety of techniques, including sessile-drop measurements [202], micropipette aspiration [203], and fluctuation spectroscopy [204]. The fluctuation-spectroscopy measurements require, in addition to tension, also bending rigidity to describe the curvature-elastic parameters of the interfaces of biomolecular condensates [204] (see Table 4). Computer simulations predict the physiologically relevant range of biomolecular condensation to lie close to the critical point marking the highest temperature for phase coexistence, such that the interface tension can be expected to follow the scaling laws characterized by the critical exponents [109]. Because electrostatic interactions are often important, the addition of salt can decrease the interface tension [205,206] (see Table 4), which has been found using optical traps to apply to the viscoelasticity as well [206]. An increased fraction of uncharged, highly branched polymers in the buffer surrounding the condensate can increase the interfacial tension [207].

**Figure 11:**
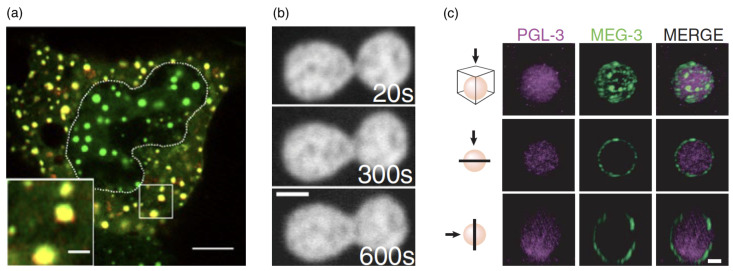
Biomolecular condensates: colocalization, coalescence, and interface decoration by protein clusters. (**a**) Cytoplasmic enhanced green fluorescent protein (EGFP)–yes-associated protein (YAP) selectively enriches different proteins. Live-cell image showing the colocalization of EGFP–YAP (green) condensates with mCherry–Dcp1a (red) condensates in the cytoplasm of HEK293T cells after hyperosmotic stress that induced condensate formation, 20s after sorbitol treatment. The dotted line indicates the nucleus, green fluorescence EGFP-YAP condensates, red mCherry-Dcp1a condensates, and yellow colocalization. The scale bars correspond to 5μm (whole-cell image) and 1μm (magnified view of the boxed region). Reproduced from Ref. [214] with permission from Springer Nature. (**b**) Time-lapse of nucleolar coalescence after the nucleolar signal at t=0s in the nucleus of a live HeLa cell. The frames show the progress of the nucleolar fusion. The scale bar corresponds to 2μm. Reprinted with permission from Ref. [209]. Copyright 2018 by the American Physical Society. (**c**) MEG-3 forms low-dynamic clusters that adsorb to the surface of PGL-3 condensates. Photomicrographs of a P granule reconstituted in vitro with purified PGL-3 and MEG-3 trace-labeled with Dylight 488 and Alexa 647, respectively. The scale bar corresponds to 3mm and applies to all images in the set. The top panels are a maximum projection of a z-stack through the granule. The middle panels are a single x-y plane through the middle of the same granule. The lower panels are a single z-x plane through the middle of the same granule. Reproduced from Ref. [20], AAAS.

Working on biomolecular condensates relevant for drug delivery can also still mean learning from nature, in particular, because the research on condensates in vivo is a very active research field as well. Condensates of different macromolecules have been found to colocalize in some cases, which may allow for an exchange of molecules (see Figure 11a). Time-lapse movies of coalescence show the droplet nature of biomolecular condensates [209], e.g., for the coalescence of nucleoli (see Figure 11b). Furthermore, their fusion and surface fluctuations allow us to measure the rheological properties of the droplet and the surrounding fluid. The viscosities of biomolecular condensates range from 100mPas to 1kPas [203]; the viscosity of nuclear speckles and nucleoli is of the order of 1–10 kPas and thus three to four orders of magnitude higher than the viscosities of many other in vitro condensates and water droplets [211]. Aging condensates have been observed to decrease in size. In parallel, their elastic modulus varies only weakly, but the viscosity increases strongly [206]. Whereas fluorescence recovery after photobleaching in PGL-3 occurs in less than a minute if the condensate is younger than 30 min, the half-life time is ≈50 min for 46-hour-old condensates. Similarly, coalescence right after formation takes place in 10s, whereas it takes tens of minutes for 46-hour-old condensates.

Interfacial protein clusters that adsorb to the surface of the condensates during LLPS have been shown to change their interfacial properties [20] (see Figure 11c). Although this is reminiscent of classical Pickering emulsions [212], the energy gain for the protein clusters to be located at the interface of a condensate is much smaller than typical energies of particles at an oil–water interface because of the smaller size of the protein clusters and the small interface tension. Therefore, although the protein clusters lower the effective interface tension and slow down coarsening as expected for classical Pickering emulsions, a condensate-cytoplasmic exchange of the clusters can be expected to lead to an equilibrium of bound ‘particles’ at the interface instead of irreversible binding. Protein cages attached to the interface of condensates can thus be used to precisely control the condensate radii [213]. Similarly, although not leading to monodisperse condensates, the binding of RNA molecules to the surface has been shown to decrease the condensate size [19].

## 3. Circulation Times

**Figure 12:**
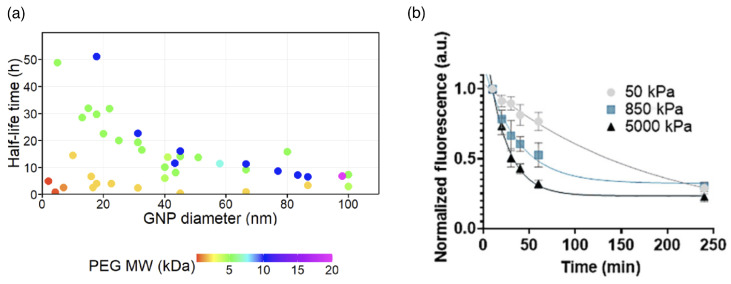
Circulation times. (**a**) Circulation half-life times of gold nanoparticles (GNP) with various diameters and PEG coatings of various molecular weights (MW). The color of the data points corresponds to the MW of the PEG coating. Adapted from Ref. [82]. CC BY 3.0. (**b**) Clearance profiles of PEG particles with diameters between 2 and 3μm and elastic moduli of 50, 850, and 5000 kPa after intravenously injecting them into mice. Reproduced from Ref. [218] with permission from Springer Nature.

The concentration of drug-delivery vectors in the blood circulation system decreases with increasing time after the administration. For small drug molecules, a characteristic time for the distribution of the drugs by permeation through the vascular wall and a significantly longer time for the elimination of the drug are distinguished. The fast and slow decrease of the concentration are also referred to as α and β phase, respectively [215]; the α phase is often found up to 1h after the administration. For polymer-based delivery vectors, the strategies to prolong the blood circulation time are versatile [216]. The elimination of the delivery vectors from the blood circulation is usually achieved by the mononuclear phagocyte system (MPS), also referred to as the reticuloendothelial system (RES). For drug-delivery vectors, often only the elimination from the circulation is characterized. A prolonged circulation time for delivery vectors without a detectable α phase is referred to as the stealth effect, whereas a prolonged characteristic time for the concentration decrease in the β phase for systems with a detectable α phase is referred to as the pseudo-stealth effect; more than 85% of stealth nanomaterials have been reported to show an α phase [215]. The half-life times of lipid–polymer hybrid nanoparticles with sizes between 100 and 200nm in the blood of mice, calculated from the measured concentration decays, can be prolonged by polymer functionalization by up to two orders of magnitude [217] (see Table 5). The half-life times of PEG-coated gold nanoparticles have been shown to increase with both increasing PEG molecular weight and decreasing gold nanoparticle size [81,82] (see Figure 12a). Studies on PEG gels with diameters of 2 to 3μm show that the circulation times increase with decreasing gel stiffness (see Figure 12b).

**Table 5:** Elimination half-life times of bare and coated liposomes and metallic (nano-)particles ^1^.

Particle	Surface Properties	Hydrodyn. Diam.	Half-Life Time $t_{1/2}$	References
lipid nanoparticles	bare	142nm	0.3h	[217]
lipid nanoparticles	HEP	164nm	1.49h	[217]
lipid nanoparticles	PEG	128nm	2.73h	[217]
lipid nanoparticles	PEG/HEP	132nm	72.6h	[217]
liposomes, first dose	bare	98.7nm	12.0h	[219]
liposomes, second dose	bare	98.7nm	12.3h	[219]
liposomes, first dose	PEG	107.7nm	33.6h	[219]
liposomes, second dose	PEG	107.7nm	1.66h	[219]
liposomes, first dose	HPMA	110.0nm	16.0h	[219]
liposomes, second dose	HPMA	110.0nm	15.5h	[219]
gold nanoparticles, 17nm diameter	2kDa PEG	25.2nm	4.0h	[81]
gold nanoparticles, 17nm diameter	10kDa PEG	63.5nm	51.1h	[81]
gold nanoparticles, 87nm diameter	10kDa PEG	118.9nm	6.6h	[81]

^1^ Experiments performed using mice.

The physicochemical properties of drug-delivery vectors are not the only parameters important for the circulation time, but also the immunological response of the organism to which the vector is applied. Bare lipid nanoparticles, liposomes, and gold nanoparticles can be eliminated from the blood circulation of mice within a few hours; polymer functionalization can extend the circulation half-life times to several days (see Table 5). However, a PEG coating can induce the production of antibodies against PEG, which significantly shortens the circulation times after a first dose has been applied. The antibodies lead to accelerated blood clearance in the liver and the spleen for follow-up applications of the vector [219,220,221,222]. Intriguingly, measurements of the half-life times of liposomes with diameters of ≈100 nm in rats showed marked differences between the first and the second dose, applied 6–8 days later, for PEG-coated liposomes. Whereas the PEG–lipid hybrid nanoparticles showed a half-life time prolonged by almost a factor of three compared with bare liposomes after the first dose, the half-life time was reduced by more than 80% compared with bare liposomes after the application of the second dose [219]. Polymers other than PEG do not induce an immunological response and thus show more promise for translation into clinical practice. For example, a difference between the half-life times after the first and second dose has not been observed for liposomes coated with, e.g., poly(vinylpyrrolidone) (PVP), poly(N,N-dimethylacrylamide) (PDMA), N-(2-Hydroxypropyl)methacrylamide (HPMA), and poly(N-acryloyl morpholine) (PAcM) [219]. Polysarcosines also show a low antigenicity [220].

For polymer-functionalized drug-delivery vectors, the functionalization changes the chemical surface properties, as well as size and elasticity. It has been shown using particle-based mesoscale hydrodynamic simulations that the size and shape of particles affect their radial distribution in capillaries, i.e., their margination at the capillary walls [223,224]. Polymer gels enable us to study the effect of elasticity on the circulation time for delivery vectors with otherwise similar physico-chemical properties and, in particular, without a need to consider the core. The longer half-life times measured for soft compared with stiff hydrogels qualitatively confirm the observed prolonged half-life times for polymer-grafted nanoparticles that are softer (and larger) than the bare particles. For PEG-based hydrogel nanoparticles with hydrodynamic radii ≈100 nm, soft particles with a bulk modulus of ≈10 kPa show an (α-phase) circulation half-life time of ≈2 h, whereas stiff particles with a bulk modulus ≈3 MPa are cleared from the circulating blood of mice within minutes [225]. However, the difference between the circulation times of both particles nearly disappears for times larger than 4h. Similarly, the organ distribution of the particles is significantly different at short times, but the differences nearly disappear after 12h when the vast majority of the particles are found in the liver and the spleen. A faster clearance of stiffer gels from the blood circulation has also been reported for PEG-based nanogels with hydrodynamic radii of ≈100 nm and elastic moduli of 37kPa and 93kPa in mice, which has been hinted to be caused by the measured faster uptake of the stiffer gels by monocytes and macrophages [226]. Similarly, polyion complex vesicles with their permeable and presumably flexible polyelectrolyte shell also show long circulation times [86,227].

## 4. Cellular Uptake Mechanisms

In vivo, erythrocytes remain in the circulation system for ≈120 days. An increased rigidity is believed to be a key factor for the elimination of old erythrocytes from the circulation. Therefore, particles to be injected into the bloodstream have been engineered with sizes, shapes, and elasticities akin to those of healthy erythrocytes, aiming to maximize the circulation time [228,229,230]. (Cargo can also be attached to erythrocytes in order to increase its circulation time [231].) Accordingly, the dependence of phagocytosis on erythrocyte stiffness has also been studied [232]. After a short discussion on measuring cellular uptake and drug release, and the role of a protein corona and polymer functionalization of drug-delivery vectors, the three main uptake mechanisms of (i) membrane translocation, (ii) passive endocytosis and fusion, and (iii) active cellular uptake processes will be discussed. Membrane translocation is particularly interesting for the delivery of proteins to cells, because it avoids endosomal trapping and allows the delivery of vectors directly to the cytosol [233,234]. Whereas endocytosis is generally considered in biology as an energy-consuming process, the term “passive endocytosis” may have been coined in chemistry or physics and is often used when the effects of particle elasticity, shape, and size, and particle–membrane adhesion for the interaction of particles with lipid-bilayer membranes are studied [235,236]. Although most cellular uptake processes do require metabolic energy, insights gained from passive endocytosis provide valuable mechanical insights that are also believed to be relevant to active uptake processes and are, therefore, also discussed in review articles on cellular uptake [237]. However, a complete mechanistic understanding of cellular uptake requires a consideration of active uptake processes also on the level of model systems.

### 4.1. Characterizing Cellular Uptake

**Figure 13:**
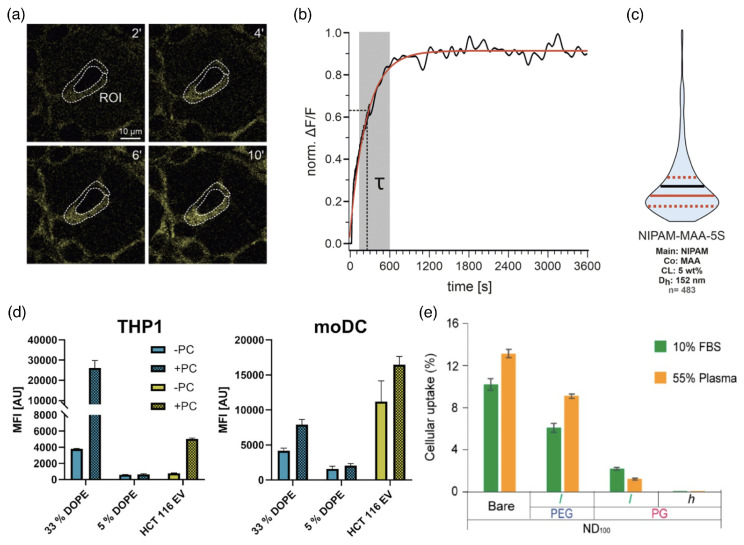
Cellular uptake. (**a**–**c**) Uptake kinetics for NIPAM-MAA-5S microgels by HEK293T cells. (**a**) Fluorescence confocal images 2, 4, 6, and 10 min after microgel application. A donut-shaped region-of-interest (ROI) demarks the cytosolic area of a representative cell. (**b**) Increase in the fluorescence in the ROI marked in (**a**). Normalized intensity changes ($\Delta F/f_{0}$) as a function of time (black trace) and as a monoexponential fit (red), used to calculate the cell-specific time constant τ. The gray background marks the experimental period represented in (**a**). (**c**) Violin plot for the distribution of time constants, starting at τ≈0s. Solid horizontal lines indicate the mean (black) and median (red) values, and the upper and lower quartiles (dashed lines). Adapted with permission from Ref. [238]. Copyright 2020 American Chemical Society. (**d**) Uptake of liposomes (blue) and extracellular vesicles (green) with and without protein corona (PC), determined using flow cytometry to measure the mean fluorescent intensities characterizing the uptake of the particles into THP1 and moDC cells after 16h. Mean values and standard deviations of median fluorescence intensities are shown (n=3). Reprinted from Ref. [53]. CC BY 4.0. (**e**) Quantification of nanodiamonds taken up by macrophages after 12h incubation time, determined using extinction spectroscopy. The data are represented as the mean value ± standard deviation of three independent replicates. Reprinted with permission from Ref. [54]. Copyright 2020 American Chemical Society.

Cellular uptake of drug-delivery vectors can be characterized in vitro using fluorescence microscopy. For example, by fitting the fluorescence intensity of HEK293T cells that are exposed to NIPAM-MAA-5S microgels with an exponential function, a characteristic timescale for cellular uptake between seconds and minutes has been found (see Figure 13a–c). Small and less crosslinked microgels are taken up faster than larger microgels and microgels with a higher degree of crosslinking [238]. However, not only the kinetics of the uptake process but also the total uptake within a specific time window is of interest for characterizing the cellular uptake of a drug. Furthermore, eventually the availability of the drug to the cell is key for therapeutic applications. Two different time scales thus have to be distinguished: for the delivery of the drug-delivery vector and for the delivery of the drug; both are determined by the vector’s structural and chemical properties, and the latter can take significantly longer [239]. Controlled in situ formation of condensates can even be used as reservoirs for therapeutics in cells [240]. However, loading and release kinetics will not be discussed in detail here.

In biological fluids, the formation of a protein corona around drug-delivery vectors is a crucial factor for cellular uptake. A study for the uptake of synthetic and extracellular vesicles with diameters of ≈100nm with and without a protein corona showed that the uptake can be significantly increased in the presence of the corona [53] (see Figure 13d). For phagocytic THP1 cells, the presence of a corona can increase cellular uptake by almost up to an order of magnitude, and for monocyte-derived moDC cells, by up to a factor of 1.5–2. Phagocytic uptake by U937 cells in fetal bovine serum (FBS) and plasma is suppressed by about 50% using a polyethylene glycol (PEG) coating and, to a much higher percentage, using a polypropylene glycol (PG) coating [54] (see Figure 13e). Polymer functionalization not only changes the sizes, shapes, and elastic properties of drug-delivery vectors but also influences the formation of a protein corona. Thus, the chemical surface properties of the delivery vectors are also crucial for understanding their uptake [54].

### 4.2. Lipid-Bilayer Translocation

**Figure 14:**
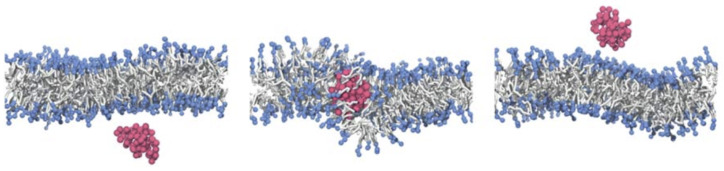
Simulation snapshots of a linear polymer chain with intermediate hydrophobicity translocating through a lipid-bilayer membrane. Used with permission of the Royal Society of Chemistry, from Ref. [63]; permission conveyed through Copyright Clearance Center, Inc.

Linear polymer chains with suitable hydrophobic/hydrophilic properties have been shown to translocate through lipid bilayers (see Figure 14). Here, a balanced hydrophobicity of each monomer or an appropriately designed sequence of hydrophilic and hydrophobic monomers allows the polymer to experience the lipid-bilayer membrane as a small perturbation to a flat potential-energy landscape, such that the polymers translocate through the membrane almost freely [63,64]. The translocation is accompanied by an increase in the permeability of the membrane to solvent, which is maximal in a finite distance in the plane of the membrane from the center of the polymer. Very hydrophobic polymers aggregate in the core of the bilayer along with the tails of the lipid molecules, whereas hydrophilic polymers cannot enter the hydrophobic core and experience the membrane as confinement [63,64].

The translocation rates for linear copolymers with a balanced periodic sequence of hydrophobic and hydrophilic monomers that repeats at distances below the thickness of the lipid bilayer are independent of the polymer chain length. In contrast, the translocation rates for random copolymers with an overall balanced hydrophobicity decrease exponentially with increasing chain length [62]. Polymer translocation may be influenced by membrane shape. Simulations using a dumbbell model for the lipid molecules in bilayers predicted asymmetric polymer transport, because of a lipid-entropy difference due to a curvature-induced difference in lipid fluctuations in the two monolayers [241]. Similarly, using a chain-like lipid model, it has been predicted that for polymers being partitioned in the hydrophobic core of the membrane, a higher concentration of polymers is expected to be found in the outer monolayer of a curved bilayer. Although the membrane-curvature dependence of polymer translocation has not yet been studied using more recent lipid models, it can be expected to be observed also for the more sophisticated coarse-grained modeling approaches that are currently used.

Alternating amphiphilic copolymers have been synthesized, and their interaction with giant unilamellar vesicles (GUVs) has been studied using fluorescence microscopy [60]. The GUVs were initially devoid of polymers. Using kinetic modeling, a fast saturation of the membrane with polymers and a slow release of the polymers to the interior of the vesicle have been predicted. The translocation rate has been shown to decrease with increasing lipid chain length and bilayer thickness, both of which suggest the presence of an increased free-energy barrier. The experimentally observed translocation of polymers through lipid bilayers is thus similar to predictions from analytical and computer-simulation studies on the translocation of linear polymers through holes/pores [242], where the free-energy barrier for translocation results from the reduced entropy of the polymer chain. Brownian Dynamics simulations demonstrated an increased free-energy barrier for pore translocation with increasing pore length [243]. Synthetic polymers with alternating hydrophilic and hydrophobic units for lipid-bilayer translocation, which can be used as drug carrier systems for targeted and controlled delivery, have been patented [61].

**Figure 15:**
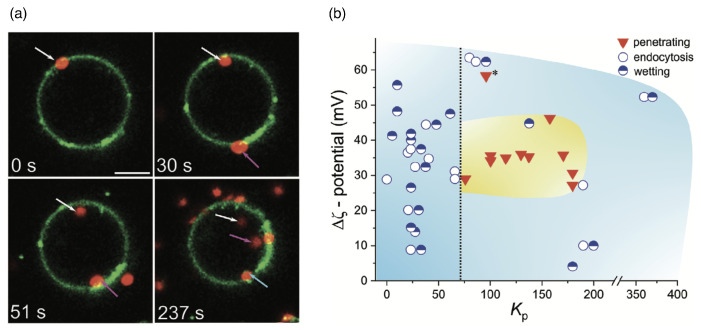
Wetting, endocytosis, and lipid-bilayer translocation of condensates at liposomes. (**a**) Translocation of condensates composed or oligoarginine ($R_{10}$, 2.7kDa) and torula yeast RNA (tyRNA) (red, labelled with DNA ${\mathrm{polyA}}_{15}$-Cy5 oligonucleotides, marked with arrows) at liposomes consisting of ${\mathrm{POPC}}_{0.4}/{\mathrm{cholesterol}}_{0.1}/{\mathrm{POPG}}_{0.5}$ (green), labelled with DOPE-AttO 488. The scale bar corresponds to 10μm. (**b**) Morphological state diagram of the interplay between complex condensates and liposomes as a function of their absolute ζ-potential difference Δζ and the condensate’s lipid partition coefficient $K_{p}$. * Denotes a special case of $R_{40}/\mathrm{polyA}$ condensates that were significantly smaller (average diameter <1μm) than most other condensate samples and were found to penetrate the liposome membrane despite a strong surface attraction. Reprinted from Ref. [113]. CC BY 4.0.

A translocation-like integration into the lipid-bilayer membrane without wrapping has, using computer simulations, also been observed beyond linear chains for nanogels [244]. However, in the latter case, the translocation process did not take place because, after attachment, the gels no longer detached from the membranes. Lipid-bilayer penetration has been experimentally suggested for polymer-coated nanoparticles [115] and has been directly observed for biomolecular condensates. It allows for a direct delivery of drugs to the cytosol without envelopment of the delivery vector by the lipid-bilayer membrane [113], as shown for condensates of oligo-arginine and DNA in Figure 15a. Electrostatic attraction, which can be tuned by the ζ potential of the condensate, mediates an attraction between the condensate and the bilayer and leads to either wetting of the membrane, endocytosis, or—for optimal values of the partitioning coefficient—membrane translocation (see Figure 15b). A decrease in the vesicle size after translocation shows that some lipids remain within the condensate. For smaller condensates, translocation has been observed for higher zeta potentials compared to the large condensate. Condensates have also been shown to enter cells [113]. Micrometre-sized pH and redox-responsive condensates have been reported to deliver small peptides, enzymes, and messenger RNAs directly to the cytosol [114].

### 4.3. Passive Endocytosis

**Figure 16:**
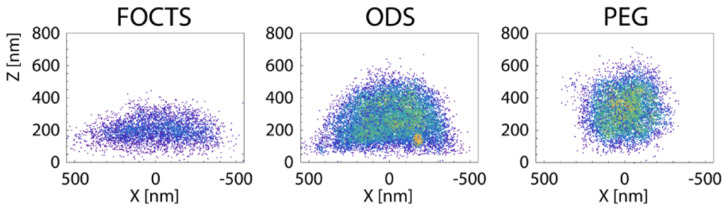
Images of direct stochastic optical reconstruction microscopy measurements of adsorbed PNIPMAM microgels at surfaces of different functionalization. The microgel conformation changes from a fried egg shape at hydrophobic surfaces coated with trichloro(1H,1H,2H,2H-perfluorooctyl)silane (FOCTS) or n-octadecyltrimethoxysilane (ODS) to a spherical shape at hydrophilic surfaces coated with PEG. The solid–liquid interfaces are placed at z=0. Reprinted with permission from Ref. [245]. Copyright 2019 American Chemical Society.

**Figure 17:**
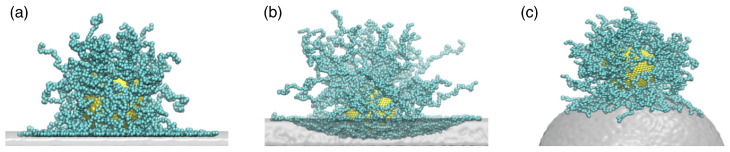
Snapshots of three systems of a PGN interacting with attractive substrates having: (**a**) zero (flat), (**b**) positive (hole/concave), and (**c**) negative (bump/convex) curvatures. Reprinted from Ref. [248]. CC BY 4.0.

Passive endocytosis is cellular uptake by membrane wrapping of the delivery vector. It requires the adhesion of the vector to the membrane, which—due to the elasticity of the material—involves a deformation of both the polymeric delivery vector and the lipid-bilayer membrane. Adhesion of initially spherical microgels to planar substrates can result in oblate, fried-egg microgel shapes and a significant decrease of both volume and surface area [245] (see Figure 16); the microgel deformation increases with increasing adhesion strength. Using computer simulations, we showed that such strong microgel deformations can be expected for initially spherical ultra-soft microgels of radius 200nm with Young’s modulus Y≈5kPa attached to lipid-bilayer membranes with bending rigidity $50\;k_{B}T$, for receptor-ligand adhesion strengths orders of magnitude below those measured for a spherical agarose bead interacting with a planar glass substrate [246,247]. Micro- and nanogel deformations when adhering to lipid-bilayer membranes are more likely to occur the smaller the gel is. Fried-egg shapes are also observed using computer simulations for the adsorption of PGNs to substrates (see Figure 17). Comparing planar and curved substrates, the simulation results show that the adhesion energy gain for identical PGNs adhering to a planar substrate is higher than on the inside or outside of spherical caps [248]. This finding assumes a homogeneous adhesion strength between the grafted linear chains and the substrate, which may not apply to all PGNs. For nano- and microgels, a homogeneous adhesion may describe the interaction of very soft gels with lipid-bilayer membranes [226]. Various other microgels, however, have also been reported to interact with substrates via discrete adhesion sites, such as dangling chains that insert into the tail region of the lipid bilayer [249], dopamine methacrylamide crosslinkers [250], and screened electrostatic interactions at physiological salt concentrations [251].

**Figure 18:**
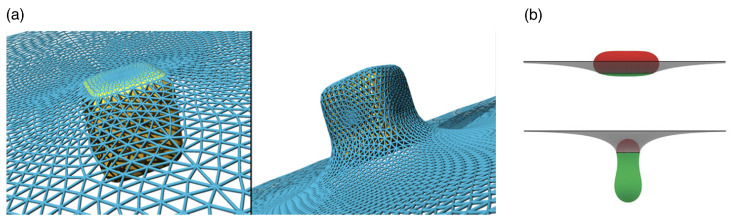
Wrapping of non-spherical particles. (**a**) Shallow- and deep-wrapped states of a cube at a triangulated membrane. Reprinted with permission from Ref. [252]. Copyright 2014 American Chemical Society. (**b**) Shallow- and deep-wrapped states of prolate vesicles: attached (green) and free (red) vesicle membrane area. Reprinted from Ref. [256]. CC BY 4.0.

Because the wrapping of polymeric particles usually involves a deformation of the particles in partial-wrapped states, insights on wrapping deformable particles may be gained from studies of wrapping non-spherical hard particles (see Figure 18). Here, highly curved regions of the particle surface correspond to wrapping-energy barriers. This leads to an increased stability of partial-wrapped states for non-spherical compared with spherical hard particles. For example, cube-like particles experience particularly stable partial-wrapped states if the wrapping of additional edges can be avoided [252]. Oblate ellipsoidal hard particles adhere to membranes with their flat side at weaker adhesion strengths than spherical particles with the same surface area, but wrapping the highly curved rim constitutes an energy barrier [253]. Only results of a few studies on model systems are available for wrapping of polymeric particles, including studies for stiff microgels at GUVs [157,249], capsules [254], and small nanogels [255]. So far, state-of-the-art theoretical and simulation studies on the wrapping of elastic particles use vesicles and vesicle-like particles, where the 2D shells determine the elastic properties, as a generic model system [254,256,257,258,259,260,261]. For initially spherical vesicles and capsules, shape transitions from spherical via oblate with the flat side attached to the membrane, prolate with the long axis perpendicular to the membrane, and back to spherical have been predicted [254,261]. Wrapping of initially prolate vesicles involves a change from a stable submarine to a stable rocket orientation as predicted for hard particles [252], in addition to a deformation of partially wrapped vesicles [256]. A shape change has also been experimentally observed for the cellular uptake of 200nm sized initially spherical elastic silica nanocapsules with Young’s moduli between 500kPa and 1.18kPa [262].

**Figure 19:**
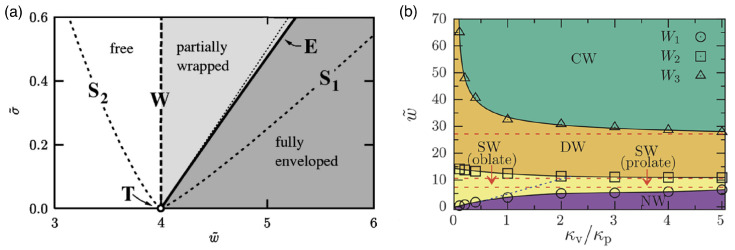
Wrapping diagrams for particles at initially planar membranes. (**a**) Diagram for a hard spherical particle in the plane of reduced adhesion strength $\tilde{w}$ and reduced membrane tension $\tilde{\sigma }$ close to the triple point T. The dashed line “W” marks the continuous transition at which partial wrapping sets in, the bold solid line “E” indicates the discontinuous transition between partially wrapped and fully enveloped, and the short dashed lines “$S_{1}$” and “$S_{2}$” are the spinodals belonging to “E”. The fine dotted line $\tilde{w}=4+2\tilde{\sigma }$ close to E indicates where the fully wrapped state has zero energy. Figure adapted with permission from Ref. [272]. Copyrighted by the American Physical Society. (**b**) Diagram for a prolate elastic particle modeled by a vesicle with reduced volume v=0.8 at a membrane with reduced tension $\tilde{\sigma }=0.5$ in the $\kappa_{v}/\kappa_{p}$-$\tilde{w}$-plane. Here, $\kappa_{v}$ is the bending rigidity of the vesicle membrane and $\kappa_{p}$ the bending rigidity of the initially planar membrane. The binding transition $W_{1}$ separates the non-wrapped (NW) from the shallow-wrapped (SW) regime, the transition $W_{2}$ the SW from the deep-wrapped (DW) regime, and the envelopment transition $W_{2}$ the DW from the complete-wrapped (CW) regime. The red-dashed lines indicate the wrapping transitions for a hard particle with reduced volume v=0.8. Reprinted from Ref. [256]. CC BY 4.0.

In general, from a mechanical point of view, the wrapping of hard particles at lipid-bilayer membranes is well understood [263,264]. Synthetic model systems [231,265,266,267], as well as theory and computer simulations [235,252,253,268,269,270,271,272] have helped to rationalize both the wrapping process and the membrane-mediated interactions between partially wrapped nano- and microparticles at membranes. Spherical hard particles at tensionless membranes directly transition from the non-wrapped to the complete-wrapped state beyond the threshold adhesion strength $w=2\kappa /(\pi R^{2})$, where κ is the bending rigidity of the membrane and *R* the particle radius [271,272]. Systematic calculations for particle wrapping show that a finite membrane tension stabilizes partial-wrapped states (see Figure 19a). If such a continuum membrane model is used for the calculations, often dimensionless parameters like the reduced adhesion strength $\tilde{w}=wA_{p}/\left(2\pi \kappa \right)$ and the reduced membrane tension $\tilde{\sigma }=\sigma A_{p}^{2}/\left(\pi \kappa \right)$ for a spherical particle help to make the numerical predictions transferable to experimental systems with various particle surface areas $A_{p}$. For prolate vesicles, calculations of wrapping states using a continuum membrane model predict a dominance of vesicle shape for high bending rigidity ratios $\kappa_{v}/\kappa_{p}$ between the vesicle membrane and the membrane the vesicles attach to, and a dominance of vesicle deformability leading to a strongly enlarged regime of stable partial-wrapped states for highly deformable vesicles and small $\kappa_{v}/\kappa_{p}$ (see Figure 19b).

For wrapping fluid biomolecular condensates, wrapping of a droplet may be a suitable model system. This system has been studied using aqueous phases of polymer solutions in vesicles, for which the importance of the difference between an intrinsic contact angle between the phases and an extrinsic contact angle observed in microscopy has been pointed out [273,274]. More recently, it has been shown that not only the condensate composition but also solution salinity [275] and membrane composition [276], such as the cholesterol content, affect the condensate–membrane interaction. The observed states are not limited to almost spherical and lens-shaped partial-wrapped condensates, but also include complete wetting of the membrane by the condensate [275,277].

### 4.4. Active Cellular Uptake Processes

**Figure 20:**
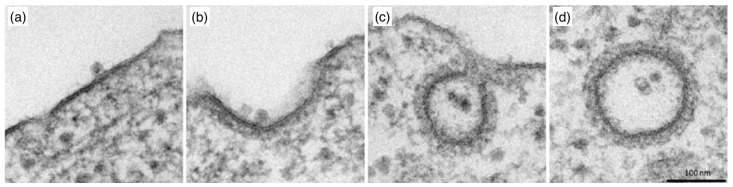
The endocytosis of the oncologic H-1 parovirus (H-1PV) is clathrin-dependent. HeLa cells were infected with H-1PV for 1h at 4 °C to allow H-1PV cell surface attachment but not entry. Cells were then shifted to 37 °C to allow H-1PV cell internalization. The cells were collected every 5min for a total of 30 min and processed for EM analysis. (**a**) At 4 °C, H-1PV particles are found attached to electro-dense (clathrin-rich) regions on the plasma membrane. (**b**) In the first 5 min after release at 37 °C, H-1PV particles are detected in early-forming clathrin-coated pits. (**c**) From 10 to 30 min, H-1PV particles moved into the cells within deeply invaginated clathrin-coated pits that were still connected to the plasma membrane, forming an hourglass-like membrane neck. (**d**) Later in the infection (10–30 min at 37 °C), H-1PV particles are seen being trafficked within the cell inside clathrin-coated vesicles. Reprinted from Ref. [278]. CC BY 4.0.

**Figure 21:**
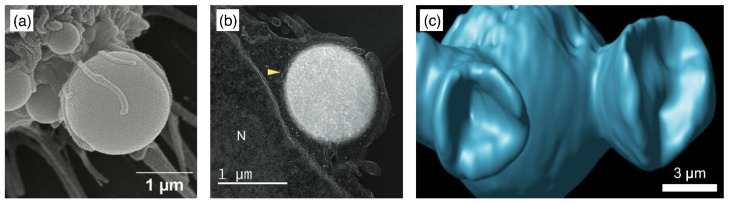
Cytoskeleton-driven active uptake processes. (**a**,**b**) Uptake of short histidine-rich, pH-responsive beak peptide (HB*pep*) coacervates by HeLa cells. (**a**) Filopodial capture visualized using scanning electron microscopy and (**b**) almost entirely engulfed HB*pep*-SP coacervates with an additional lysine residue, visualized via ultrathin sections of fixed and resin-embedded cells 15min after incubation. The yellow arrow indicates the membrane around the condensate, and N the nucleus. Reprinted from Ref. [112]. CC BY 4.0. (**c**) Macropinocytic cups of dictyostelium obtained from a 3D-rendered fluorescent image. Reprinted from Ref. [286]. CC BY 4.0.

Biological cells possess the machinery to take up drug-delivery vectors using active, metabolically driven processes. For example, cargo with sizes of ≈100 nm diameter can be taken up using clathrin-mediated endocytosis [278,279] (see Figure 20). Here, the binding of a clathrin coat assists the budding of the cell’s plasma membrane and, therefore, cellular uptake. For cargo with sizes larger than ≈500 nm, active cellular uptake is mainly driven by active cytoskeletal processes [280,281], reported as early as 1967, where it was observed for the uptake of clusters of latex beads by Acanthamoeba [282]. For single particles, cytoskeleton-driven cellular uptake is most efficient for particle sizes of 2–3 μm diameter [79,80]. Phagocytosis—by phagocytes that are specialized for this process (e.g., macrophages and neutrophils)—is usually associated with solid, stiffer cargo [7,283,284,285]. Macropinocytosis, via macropinocytic cups, is not tied to a specific cell type and is usually associated with fluid cargo [286]. Recently, the uptake of micrometer-sized viscoelastic, glassy biomolecular condensates has been observed to integrate features of macropinocytosis and phagocytosis [112] (see Figure 21). The condensates have been shown not to translocate through the lipid bilayer using GUVs, and molecular inhibitors confirmed the role of active cytoskeletal remodeling in cellular uptake. After an incubation time of 15min, some HB*pep* condensates were already attached to HeLa cells and surrounded by filopodia, whereas HB*pep*-SB condensates were almost completely engulfed. Furthermore, condensates “sinking” into the cells have been observed.

Data for several phagocytic cells suggest that stiffer particles are taken up faster, with a threshold Young’s modulus beyond which cells recognize particles as hard [287]. Neutrophils, as a special case, have been found to effectively phagocytose elastic particles with a wide range of stiffnesses [288]. However, particle elasticity also affects other active cellular uptake pathways and may even be a decisive factor in determining which uptake pathway a cell uses. For example, hard hydrogel nanoparticles have been observed to be taken up by clathrin-mediated endocytosis, soft ones by macropinocytosis [6]. A different study shows that hard 100nm particles are taken up by clathrin and caveolae-mediated endocytosis, while soft ones are taken up—much faster—by caveolae-mediated endocytosis and non-receptor-mediated endocytosis [289]. For tumor cells, a greater uptake of soft nanolipogels (Y<1.6MPa) over hard nanolipogels (Y>13.8MPa) has been reported, leading to the soft gels being deposited in the tumor and hard gels in the liver [99]. However, for polymeric drug-delivery vectors, the interactions with cells in vitro have been found to differ both by the elasticity of the carrier and the cell type, as shown in Ref. [290]. Whereas circulation times have consistently been found to be prolonged with increasing particle deformability, ambiguous results are reported for the dependence of cellular uptake by immune cells, cancer cells, and endothelial cells.

Active cellular uptake involves complex biological signalling and regulation, a detailed discussion of which is beyond the scope of this review article. Unlike for several passive uptake mechanisms, the mechanistic details of the active cellular uptake processes are often not yet well understood. A more detailed discussion of active cellular uptake processes can be found in Ref. [291] for clathrin-mediated endocytosis, in Ref. [292] for caveolae-mediated endocytosis, in Refs. [293,294,295] for phagocytosis, and in Refs. [293,296,297,298] for macropinocytosis.

## 5. Polymers In Vivo Relevant to Drug Delivery

Polymeric materials are not only used for designing synthetic drug-delivery vectors. Upon cellular uptake, the drug-delivery vectors and the drugs themselves interact with both the lipid-bilayer membranes and the polymeric materials in vivo, which affect cellular uptake and drug delivery. Examples include polymeric layers that act as barriers, such as the mucus in the lung [299], the mucous layer in the eye [300], the pericellular coat of chondrocytes [126], and the spectrin cytoskeleton of erythrocytes [301]. This may hinder cellular uptake. Biomolecular condensates, on the contrary, may serve as natural intracellular reservoirs for drugs. For example, it has been shown in vitro that antineoplastic drugs accumulate in specific condensates driven by physicochemical mechanisms independent of the drug target [302]. However, the subcellular distribution of small molecules in cells is condensate- and also cell-state-dependent; the drug sunitinib, which is used for cancer treatment, concentrates in nucleoli in interphase cells and in condensed chromatin during mitosis [303]. Therefore, in vivo, polymeric materials can act as barriers hindering the drug delivery, but in the form of intracellular biomolecular condensates, they can also directly regulate the concentration and activity of the drugs that have already been delivered to cells.

## 6. Summary

Polymeric materials are highly versatile and have been extensively studied both experimentally and theoretically. Plasma membranes of biological cells are lipid bilayers that serve as communication interfaces, allowing cells to exchange signals and materials with their environment. For applications as drug-delivery vectors, the polymeric materials have to cross the plasma membranes. In vivo and in vitro experiments quantify key observables, such as the circulation time and cellular uptake. Computer simulations help us to connect the molecular structures of delivery vectors with appropriate models on the scale of entire vectors. Here, coarse-grained and continuum models focusing on the key parameters that describe the elastic properties of polymeric drug-delivery vectors are more appropriate for building a systematic understanding of the relationship between delivery vectors and membranes than—usually computationally also unfeasible on the relevant scales—atomistic models.

Prominent architectures of polymeric and polymer composite drug-delivery vectors are linear chains, star polymers, polymer-grafted nanoparticles, polymersomes and stealth liposomes, nano- and microgels, and biomolecular condensates. Whereas linear polymer chains in good solvents have elongated shapes, star polymers, polymer-grafted nanoparticles, polymersomes, stealth liposomes, and nano- and microgels are often spherical colloids. The origins of their elastic properties are fundamentally different for the various architectures. For example, star polymers can be considered as fluid droplets because the arms can freely reorient; this fluidity is decreased for polymer-grafted nanoparticles, particularly for large core sizes and short polymers. Microgels have a finite shear modulus and can be modeled using continuum models with 3D elastic moduli. Fluid condensates can be modeled as droplets with an interface tension and bending rigidity determining their elasticity. Intriguingly, polymeric materials can be engineered to change size, shape, and elastic properties upon exposure to external stimuli, such as changes in temperature and pH.

Using vesicles as elastic model particles, the effects of particle deformability on wrapping and passive endocytosis have been systematically predicted. However, the complex molecular interactions between polymeric drug-delivery vectors and lipid-bilayer membranes are not entirely captured by such generic elastic-particle models. For example, the deformation energy of a gel with 3D elasticity scales differently with the particle size than an elastic shell with 2D elasticity. Furthermore, for linear chains, the importance of having appropriate hydrophobic–hydrophilic properties to allow them to translocate through lipid bilayers has been demonstrated. Therefore, considering the various specific structural and chemical properties—and, in particular, the origin of the elasticity—is required to understand the cellular uptake of polymeric delivery vectors.

## 7. Outlook

Combining information on the structural and elastic properties of drug-delivery vectors with quantitative experimental data on cellular uptake may in the future lead to an improved physico-chemical-informed design of polymeric and polymer-functionalized vectors. Polymer functionalization of materials also helps us to control their interactions with biological fluids, the formation of a protein corona, and thus the molecular interactions between drug-delivery vectors and biological cells.

The elastic properties of polymeric and polymer-functionalized materials can be tuned in a wide range of elasticities that are comparable to the elasticities of eukaryotic cells [304]. However, from the modeling point of view, our understanding of specific properties of polymeric materials in general is much more advanced than our understanding of their cellular uptake. For example, even for the passive wrapping of polymeric colloids with different architectures at lipid-bilayer membranes, such as microgels and star polymers, extensive mechanistic studies are missing. Another specific example is composite materials, such as condensates, which can encapsulate and deliver a large variety of cargo to cells. Cytoskeletal remodeling has been shown to be crucial for their active cellular uptake, and stiffer particles are usually taken up more readily. Furthermore, direct cytosolic delivery has been reported. Although the cellular uptake of microgels can be hypothesized as the wrapping of a soft particle with 3D elasticity, the observation that small and less crosslinked microgels are taken up significantly faster may also hint at translocation as a potential alternative uptake pathway [238]. Only very few mechanistic models address active cellular uptake mechanisms. A thorough understanding of several passive and most active uptake pathways—and what determines which uptake pathway is dominant—is thus still lacking for most delivery vectors. Systematic studies of specific delivery mechanisms for specific vectors may help to more effectively design polymeric drug-delivery vectors in the future.

Polymeric drug delivery vectors are unique because they can change their physico-chemical properties upon exposure to external stimuli. Magnetically responsive condensates, for example, can be utilized in an alternating magnetic field for both controlled drug delivery and local heating [305]. Stimulus sensitivity can also help recruit and release drugs, as shown, e.g., for cargo recruitment by condensates triggered by temperature, pH, and ionic strength [306]. For drug loading, various methods including laser-induced phase transitions are available [307]. Within a cell, mainly a chemical sensitivity to pH or small molecules, such as condensate-modifying drugs [308], can be exploited to trigger drug release [309,310]. The stimulus sensitivity of physicochemical properties may also be important for modulating the vector–membrane interaction and, thus, cellular uptake. Furthermore, apart from direct cytosolic delivery, it is crucial to enable the drugs to escape from endosomes and lysosomes to the cytosol and make them available to the cells. Endosomal and lysosomal escape can be triggered by exploiting the pH responsiveness of polymeric materials [311,312,313]; for example, delivery vectors can swell or disintegrate in the acidic conditions found in lysosomes [312]. Condensates that lead to lysosome enlargement and permeability can even assemble only intracellularly [314]. Currently, the large variety of exciting possibilities that the stimulus-sensitivity of polymeric materials offers for applications in drug delivery awaits further characterization.

Finally, so far, most systematic mechanistic studies of model systems assume an interaction of drug-delivery vectors with lipid-bilayer membranes only. Although this provides important insights on vectors crossing the plasma membrane, in vivo, plasma membranes may have a polymer coat and be supported by a cortical cytoskeleton. Nuclear pore complexes, a gateway for macromolecules to the cell’s nuclei, are decorated with polymeric nucleoporin structures. The implications of the interplay of these in vivo polymeric structures with polymeric drug-delivery vectors also require a systematic and quantitative bottom-up characterization.

## Figures and Tables

**Table 1:** Typical sizes of polymeric and polymer-functionalized particles.

Particle	Particle Size $R_{p}$	References
star polymers	3–40 nm	[12,13,14,15]
diblock-copolymer micelles	25–100 nm	[16,17]
biomolecular condensates	5nm–5 μm	[18,19,20]
nanogels	10–100 nm	[21]
polymer-grafted nanoparticles	20–500 nm	[22,23,24,25]
polymersomes ^1^	20nm–200μm	[26]
DNA and RNA origami particles	30–400 nm	[27,28,29,30]
stealth liposomes	200nm–1μm	[31]
microgels ^1^	100nm–100 μm	[21,32]

^1^ Although polymersomes and microgels with sizes of the order of 100μm are reported in the literature, for drug delivery by cellular uptake, only particles small enough to be taken up by biological cells are relevant.

**Table 2:** Persistence lengths of polymers in water.

Polymer	Persistence Length $\ell_{p}$	References
polyethylene oxide (PEO)	0.37–0.48 nm	[35,36]
polyethylene glycol (PEG)	0.38nm	[36]
poly(N-isopropylacrylamide) (PNIPAM)	0.25–1.5 nm	[37,38]
single-stranded RNA and DNA	1–3 nm	[39,40]
spectrin tetramers	7.5nm	[41]
double-stranded RNA and DNA ^1^	50–65 nm	[42,43]
collagen	14–180 nm	[44]
intermediate filaments	0.5–2 μm	[45]
filamentous actin	17.7μm	[46]
microtubules ^2^	80–5000 μm	[47]

^1^ Persistence length measured under physiological conditions. ^2^ Microtubules show a length-dependent persistence length [47].

**Table 3:** Elastic moduli of nano- and microgels ^1^.

Microgel	Young’s Modulus *Y* (Swollen)	*Y* (Collapsed)	References
${\mathrm{PAAm}}_{\mathrm{BIS}}$	6.5±1.6kPa	n/a	[146]
POx-HASH	7.1±2.5kPa	n/a	[146]
${\mathrm{PNIPAM}}_{\mathrm{SCL}}$	13±3kPa	n/a	[142]
$P{\left({\mathrm{OEGMA}}_{300}\right)}_{\mathrm{SCL}}$	123±6kPa	n/a	[142]
PNIPAM with 20% AAc	≈150 kPa	≈400 kPa	[158]
PNIPAM with BIS ^2^	0–1000 kPa	20 times increased	[159]
PNIPAM with BIS and DMA ^2^	0–1000 kPa	>100 times increased	[159]
$P({\mathrm{MEO}}_{2}\mathrm{MA}$-co-$OEGMA_{300}{)}_{SCL}$	292±2kPa	n/a	[142]
$P({\mathrm{MEO}}_{2}\mathrm{MA}$-co-$OEGMA_{500}{)}_{SCL}$	850±6kPa	n/a	[142]
$P({\mathrm{MEO}}_{2}\mathrm{MA}$-co-$OEGMA_{500}{)}_{EGDMA}$	63,000 ±500kPa	n/a	[142]

^1^ The microgels with Young’s moduli Y>10kPa have radii in the range $200\;\mathrm{nm}\leq R_{\mathrm{mg}}\leq 600\;\mathrm{nm}$, relevant to drug delivery, wguke those with Y<10kPa have radii $12\;\mu m\leq R_{\mathrm{mg}}\leq 15\;\mu m$.^2^ Data for various concentrations of the crosslinker BIS and the cononomer DMA.

**Table 4:** Interface tensions and bending rigidities of biomolecular condensates.

Condensate	Interface Tension γ	Bending Rigidity κ	References
clotrimazole	0.627μN/m	$2.05\;k_{B}T$	[204]
FXR1 overexpression and sodium arsenide	0.741μN/m	$1.36\;k_{B}T$	[204]
sodium arsenite	1.26μN/m	$2.08\;k_{B}T$	[204]
PGL-3 proteins at various KCl concentrations ^1^	1–5 μN/m	n/a	[208]
nucleoli in HeLa cell nuclei	1.5±0.5μN/m	n/a	[209]
$\mathrm{Ddx}4^{N}$ 1–231 in aqueous buffer	38±3μN/m	n/a	[202]
ELF3 proteins	49±9μN/m	n/a	[210]
polylysine:heparin mixture and Ficoll70 ^2^	70–125 μN/m	n/a	[207]
$\mathrm{Ddx}4^{N}$ 1–229 in aqueous buffer	82±10μN/m	n/a	[202]
LAF-1 RGG	159±10μN/m	n/a	[203]
${\left[\mathrm{RGRGG}\right]}_{5}$-dT40 at various NaCl concentrations ^1^	0.5–1.6 mN/m	n/a	[205]

^1^ Higher surface tensions are measured at lower salt concentrations. ^2^ Equimolar mixture of polylysine and heparin, for various Ficoll70 concentrations.

## Data Availability

No new data were created or analyzed in this study.

## Abbreviations

The following abbreviations are used in this manuscript:

AFM	Atomic force microscopy
BIS	N,N′-methylenebis(acrylamide)
DMA	Dopamine methacrylamide
DNA	Deoxyribonucleic acid
DOPE	1,2-Dioleoyl-sn-glycero-3-phosphoethanolamine
FOCTS	Trichloro(1H,1H,2H,2H-perfluorooctyl)silane
GNP	Gold nanoparticles
GUV	Giant unilamellar vesicle
HEP	Heparin
HPMA	N-(2-Hydroxypropyl)methacrylamide (HPMA)
LCST	Lower critical solution temperature
LLPS	Liquid–liquid phase separation
MPS	Mononuclear phagocyte system
ODS	N-octadecyltrimethoxysilane
PAcM	Poly(N-acryloyl morpholine)
PDMA	Poly(N,N-dimethylacrylamide)
PEG	Polyethylene glycol
PEO	Polyethylene oxide
PG	Polypropylene glycol
PGN	Polymer-grafted (hairy) (nano-)particles
PNIPAM	Poly(N-isopropylacrylamide)
PNIPMAM	Poly(N-isopropylmethacrylamide)
POPC	1-palmitoyl-2-oleoyl-sn-glycero-3-phosphocholine
POPG	1-Palmitoyl-2-oleoyl-sn-glycero-3-phosphoglycerol
PVP	Poly(vinylpyrrolidone)
RES	Reticuloendothelial system
RNA	Ribonucleic acid
SCL	Self-crosslinked
UCST	Upper critical solution temperature
ULC	Ultra-low crosslinked
VPTT	Volume phase transition temperature
WCA	Weeks–Chandler–Andersen
